# Deep Learning for Brain MRI Confirms Patterned Pathological Progression in Alzheimer's Disease

**DOI:** 10.1002/advs.202204717

**Published:** 2022-12-27

**Authors:** Dan Pan, An Zeng, Baoyao Yang, Gangyong Lai, Bing Hu, Xiaowei Song, Tianzi Jiang, Alzheimer's Disease Neuroimaging Initiative (ADNI)

**Affiliations:** ^1^ School of Electronics and Information Guangdong Polytechnic Normal University Guangzhou 510665 China; ^2^ Faculty of Computers, Guangdong University of Technology Guangzhou 510006 China; ^3^ Department of Radiology The Third Affiliated Hospital of SUN Yat‐sen University Guangzhou 510630 China; ^4^ Clinical Research Centre Surrey Memorial Hospital Fraser Health Surrey British Columbia V3V 1Z2 Canada; ^5^ Brainnetome Center & National Laboratory of Pattern Recognition Institute of Automation Chinese Academy of Sciences Beijing 100190 China

**Keywords:** Alzheimer's disease, deep learning, longitudinal trajectories of neurodegeneration, structural magnetic resonance imaging

## Abstract

Deep learning (DL) on brain magnetic resonance imaging (MRI) data has shown excellent performance in differentiating individuals with Alzheimer's disease (AD). However, the value of DL in detecting progressive structural MRI (sMRI) abnormalities linked to AD pathology has yet to be established. In this study, an interpretable DL algorithm named the Ensemble of 3‐dimensional convolutional neural network (*Ensemble 3DCNN*) with enhanced parsing techniques is proposed to investigate the longitudinal trajectories of whole‐brain sMRI changes denoting AD onset and progression. A set of 2369 T1‐weighted images from the multi‐centre Alzheimer's Disease Neuroimaging Initiative and Open Access Series of Imaging Studies cohorts are applied to model derivation, validation, testing, and pattern analysis. An *Ensemble‐3DCNN*‐based *P*‐score is generated, based on which multiple brain regions, including amygdala, insular, parahippocampal, and temporal gyrus, exhibit early and connected progressive neurodegeneration. Complex individual variability in the sMRI is also observed. This study combining non‐invasive sMRI and interpretable DL in detecting patterned sMRI changes confirmed AD pathological progression, shedding new light on predicting AD progression using whole‐brain sMRI.

## Graphical abstract

A novel neuroimaging biomarker (*P*‐score) is derived using interpretable 3‐dimensional deep‐learning ensemble model from brain structural magnetic resonance imaging. The *P*‐score facilitates verifying patterned progressive degeneration of Alzheimer's disease (AD) and providing additional information to enrich the long‐established neuropathologic understanding of AD. The study sheds new light on predicting individual progression by integrating more detailed changes in the whole brain.

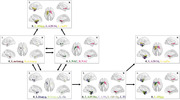

## 1 Introduction

Neurodegeneration in sporadic Alzheimer's disease (AD)^[^ ^1^ ^]^ is characterized by progressive brain atrophy and other structural changes. As a remarkable contribution to the neuropathology of AD, Braak and Braak^[^ ^2^ ^]^ have defined the progressive development of brain atrophy in AD, which has been used as the gold standard for AD staging. Based on Braak 1991,^[^ ^2^ ^]^ brain atrophy begins in basal portions of the isocortex (Stages I–II). Isocortical association area and hippocampal formation are then gradually involved (Stages III–IV). In the late phase (Stages V–VI), amyloid and neurofibrillary tangles (NFTs) spread throughout the whole brain, and the whole‐brain atrophy is developed further. Current advancements^[^ ^3^, ^4^, ^5^, ^6^ ^]^ have linked these neuropathological changes to the clinical expression of AD to assist AD staging and diagnosis on the clinical side. Multiple biomarkers, such as amyloid and tau,^[^ ^7^ ^]^ cerebro spinal fluid (CSF),^[^ ^8^ ^]^ and plasma,^[^ ^3^, ^6^, ^9^ ^]^ have been explored to allow the detection of AD. The examination of these biomarkers has implications for promoting early intervention and prevention of AD. Among these examination methods,^[^ ^3^, ^4^, ^5^, ^6^, ^7^, ^8^, ^9^, ^10^, ^11^, ^12^, ^13^ ^]^ the neuroimaging methods based on in vivo structural magnetic resonance imaging (sMRI) is promising because it can be completely non‐invasive and clinically easily available, and thus repetitively used in monitoring disease progression in the brain.

Deep learning (DL) has been utilized to analyze brain magnetic resonance imaging (MRI) images in the past decade.^[^ ^14^ ^]^ Current research has shown the expert‐level performance of DL models^[^ ^14^, ^15^, ^16^, ^17^ ^]^ in differentiating MRI images of AD subjects from those of the healthy controls (HC). In particular, the deep‐learning models^[^ ^17^, ^18^ ^]^ benefit from the strong capabilities in representation learning to effectively explore the nonlinearities in neuroimaging data. Nevertheless, most of the current DL models are developed to assist AD diagnosis. Aiming to further improve AD classification, researchers are mainly designing DL models, which are increasingly prone to complexity. Meanwhile, the difficulty in interpreting DL models becomes more remarkable with the increase in model complexity. DL models are essentially hard to explain and understand. Yet, analyzing the progression pattern of AD requires not only the models with good generalization capabilities but the interpretation techniques of models. Consequently, how to better balance the two conflicting requirements directly results in the fact that analyzing the progression pattern of neurodegeneration for neuroimaging remains challenging.^[^ ^19^ ^]^ The situation might be a reason why little attention has been paid to analyzing patterned pathological progression in AD based on DL models rather than targeting diagnostic classification tasks.^[^ ^20^, ^21^, ^22^, ^23^ ^]^ Some studies have started to extract atrophy features^[^ ^24^ ^]^ or patterns^[^ ^25^ ^]^ from sMRI to derive the brain age. In line with this effort, our recent study^[^ ^26^ ^]^ has developed a DL model (2‐dimensional convolutional neural network) to identify the critical discriminative brain regions for AD recognition. Multiple brain regions, such as the rostral hippocampus, medial and lateral amygdala, and parahippocampal gyrus, are acquired and verified as areas significantly atrophied in AD. Although our DL method using an independent training strategy and a voting‐ensemble technique^[^ ^26^ ^]^ might support the associations between neuroimaging phenotype and AD neurodegenerative progression, the longitudinal trajectory of sMRI changes during AD progression, for example, the spatial and temporal associations among neurodegenerative brain regions, were not examined in the previous study.^[^ ^26^ ^]^

In this literature, we are motivated to tackle the gap mentioned above by applying an interpretable DL technique named the ensemble of 3‐dimensional convolutional neural network (*Ensemble 3DCNN)*, which uses multiple 3D convolutional networks and a meta‐classifier to assess the degree of neurodegeneration in the brain of AD subjects. *Ensemble 3DCNN* is detailed in the Experimental Section. We derive a new neuroimaging biomarker named *P*‐score from the AD predictive scores of sMRI images, that is, the output of the trained *Ensemble 3DCNN* with sMRI images as the input. Details about the derivation of *P*‐score are reported in the Experimental Section. This neuroimaging biomarker enables the subtle detection of critical neurodegenerative changes in the AD brain using longitudinal (multiple‐time‐point) sMRI sequences acquired from the Alzheimer's Disease Neuroimaging Initialtie (ADNI, https://adni.loni.usc.edu) database^[^ ^27^ ^]^ and the Open Access Series of Imaging Studies (OASIS, https://www.oasis‐brains.org) database.^[^ ^28^ ^]^

Our objectives are to address the following questions: 1) Can interpretable DL algorithms help verify the patterned neurodegenerative changes in AD progression from the perspective of neuroimaging? 2) Can DL findings verify the progress of AD as identified in Braak 1991?^[^ ^2^ ^]^ 3) Can DL provide additional helpful information and/or critical details that the long‐established neuropathologic classics do not mention or disclose at a fine‐grained level for understanding AD? Insights toward these questions are of explicit significance. The success in verifying disease progression using DL and non‐invasive and repetitive MRI facilitates stimulating in vivo individualized staging of patients with AD. From the clinical perspective, the DL‐extracted neuroimaging biomarker provides means to track and predict the involvement sequence of neurodegenerative brain regions of patients with AD and their individual imminent clinical manifestations, which facilitates disease surveillance and prevention.

This work utilizes the large well‐established cohorts from both the ADNI^[^ ^27^ ^]^ https://adni.loni and the OASIS.^[^ ^28^ ^]^ They have advantages in terms of relatively large sample size and high generalization with data from multicenter trials. Using sMRI images, the analysis of neurodegeneration patterns in AD is conducted on the basis of the Brainnetome Atlas,^[^ ^29^ ^]^ in which the human brain is parceled into 246 fine‐grained regions according to the anatomical and functional connections.

## 2 Results

### 2.1 Participants and Datasets

Data used in this study are obtained from the ADNI database and the OASIS database. The ADNI was launched in 2003 as a public‐private partnership, led by principal investigator, Michael W. Weiner, MD. The primary goal of ADNI has been to test whether serial MRI, positron emission tomography (PET), other biological markers, and clinical and neuropsychological assessment can be combined to measure the progression of mild cognitive impairment (MCI) and early AD. The OASIS is a project aimed at making neuroimaging data sets of the brain freely available to the scientific community to facilitate discoveries in basic and clinical neuroscience.

**Figure 1:**
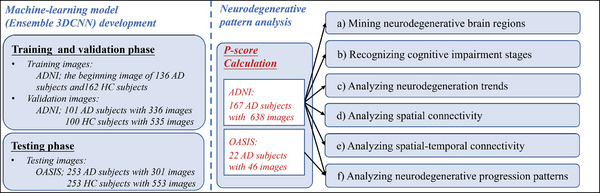
The main work of this study.

As summarized in **Figure** **1**, the main work of this study is divided into two parts, that is, deep‐learning model (*Ensemble 3DCNN*) development and neurodegenerative pattern analysis. In the first part, we accomplish three phases: the model derivation, validation, and testing. And then, after a satisfactory model is constructed, the *P*‐scores are calculated. Finally, based on the calculated *P*‐scores, the neurodegenerative pattern analyses, including six phases, that is, a) mining neurodegenerative brain regions, b) recognizing cognitive impairment stages, c) analyzing neurodegeneration trends, d) analyzing spatial connectivity, e) analyzing spatial‐temporal connectivity, and f) analyzing neurodegenerative progression patterns, are conducted in the second part. Specifically, we first investigate the neurodegenerative brain regions in each AD MRI image. Second, the cognitive impairment stage is identified. Third, while only space is fixed in longitudinal neuroimaging studies, the approximate monotonicity in *P*‐scores with varying time points is examined for each neurodegenerative brain region. Fourth, when only the time point is fixed in longitudinal neuroimaging studies, the spatial connectivity across neurodegenerative brain regions is analyzed. Fifth, while neither space nor time is fixed in longitudinal neuroimaging studies, the spatial‐temporal connectivity among the neurodegenerative brain regions across available time points is studied. Sixth, multiple progression trajectories of structural neurodegeneration across brain regions, that is, common sequential patterns of brain region degeneration, are acquired, tested, and compared with the reported patterns in Braak 1991.^[^ ^2^ ^]^ The data sets involved in all the phases are detailed as follows.

In the part of deep‐learning model (*Ensemble 3DCNN*) development, the 298 and 201 non‐overlapping subjects (including AD and HC subjects) are enrolled from the ADNI database^[^ ^27^ ^]^ for the model (*Ensemble 3DCNN*) training and validation, respectively. Among them, most subjects underwent repeated sMRI examinations at multiple time points to form a longitudinal image sequence with mainly ≈3–6 time points (i.e., sMRI images). In total, 1284 *(AD:482; HC:802)* and 871 *(AD:336; HC:535)* sMRI images are included in the training and validation datasets, respectively. In the training and validation phase, the *Ensemble 3DCNN* is trained and validated with the beginning sMRI image of each training subject *(AD:136; HC:162)* and the beginning sMRI image of each validation subject *(AD:101; HC:100)* retrieved in the ADNI database, respectively. The performance of a trained *Ensemble 3DCNN* for AD prediction is examined on the testing dataset containing the beginning sMRI image of each testing subject *(AD:253; HC:253)* retrieved in the OASIS database,^[^ ^28^ ^]^ another AD neuroimaging database. The performance of a trained *Ensemble 3DCNN* for AD prediction is detailed in the Supporting Information (i.e., Supplementary Material). We also provided the comparison results with other algorithms as Supporting Information. The demographic and health‐related information of participants in the experiments is summarized in **Table** **1**.

**Table 1:** Demographic and health‐related information of the subjects included in the studied datasets. (The listed information is collected at the beginning MRI examination of each subject retrieved in the databases.)

Dataset	Database	Category	Number of subjects (Male/Female | Right_*H*_/Left_*H*_/Any_*H*_)	Age [years]	Weight [kg]	MMSE	CDR	GDS
Training	ADNI [27]	AD	66 / 70 | 124 / 12 / ‐	76.0 ± 7.3	70.7 ± 13.8	23.2 ± 2.0	0.75 ± 0.25	1.60 ± 1.34
		HC	86 / 76 | 153 / 9 / ‐	76.3 ± 5.4	73.8 ± 13.6	29.2 ± 1.0	0.00 ± 0.00	0.80 ± 1.08
Validation	ADNI [27]	AD	61 / 40 | 88 / 13 / ‐	74.3 ± 7.8	76.0 ± 15.9	23.4 ± 2.4	0.87 ± 0.31	1.71 ± 1.51
		HC	45 / 55 | 91 / 9 / ‐	73.4 ± 5.7	76.2 ± 15.7	28.9 ± 1.3	0.00 ± 0.00	0.83 ± 1.34
Testing	OASIS [28]	AD	129 / 124 | 227 / 21 / 5	75.5 ± 7.6	81.3 ± 17.0	23.3 ± 5.3	0.82 ± 0.49	3.64 ± 2.48
		HC	156 / 97 | 224 / 22 / 7	67.0 ± 9.4	85.3 ± 18.2	29.1 ± 1.2	0.00 ± 0.00	4.24 ± 2.68

Left_*H*_, left‐handed; Right_*H*_, right‐handed; Any_*H*_, ambidextrous;

MMSE, mini mental state examination;^[^ ^30^ ^]^ CDR, clinical dementia rating;^[^ ^31^ ^]^ GDS, geriatric depressions scale.^[^ ^32^ ^]^

Note: The age, weight, MMSE, CDR, and GDS are reported as mean ± std.

**Table 2:** Statistical information on subjects and their sMRI images involved in different phases of this study

Dataset	Category\ # of images	No. of subjects with various no. of sMRI images in his/her longitudinal image sequence													No. of sMRI images	
		1	2	3	4	5	6	7	8	9	10	11	Total	⩾2	Total	Criterion 2
Training	AD	14	13	30	65	5	1	3	5	–	–	–	136	122	482	468
(ADNI [27]*)*	HC	7	10	13	27	51	33	7	4	7	2	1	162	–^a)^	162	–
Validation	AD	15	16	12	42	13	2	–	1	–	–	–	101	86	336	321
(ADNI [27])	HC	5	1	7	17	23	33	2	4	3	2	3	100	–^a)^	535	–
AD for	w/o any criterion	29	29	42	107	18	3	3	6	–	–	–	237	208	818	789
Analysis	Criterion 1	24	25	37	96	16	3	3	4	–	–	–	208	184	720	696
(ADNI [27])	Criterion 1 and 3	0	22	33	86	16	3	3	4	–	–	–	167	167	638	638
	AD (w/o any criterion)	208	42	3	–	–	–	–	–	–	–	–	253	45	301	93
Testing	AD (Criterion 1)	155	24	2	–	–	–	–	–	–	–	–	181	26	209	54
(OASIS [28])	AD (Criterion 1 and 3)	155	20	2	–	–	–	–	–	–	–	–	177	22	201	46
	HC	102	76	38	17	7	9	4	–	–	–	–	253	–^(a)^	553	–

Criterion 1: *Ensemble 3DCNN* correctly classifies all images in the longitudinal image sequence of a subject;

Criterion 2: The longitudinal image sequence contains the sMRI images acquired at no less than two time points;

Criterion 3: At least one brain region is labeled as degeneration via the neuroimaging biomarker, that is, *P*‐score, among all images in the longitudinal image sequence of a subject.

a) For the 162, 100, and 253 HC subjects in the training, validation, and testing datasets, respectively, shown in Table 1, no longitudinal image sequence has been utilized in pattern analysis phases of this study.

**Table 3:** Frequent item sets in the neurodegenerative brain regions of the 638 AD sMRI images

Rank	Item set	Frequency	Rank	Item set	Frequency
1	*{ L.mAmyg }*	82.60%	11	*{ L.vId/vIg, L.lAmyg }*	68.50%
2	*{ L.NAC }*	80.72%	12	*{ L.vIa, L.vId/vIg, L.lAmyg }*	68.50%
3	*{ L.mAmyg, L.NAC }*	79.00%	13	*{ L.TI }*	67.87%
4	*{ L.lAmyg }*	73.20%	14	*{ L.TI, L.vIa }*	67.87%
5	*{ L.vIa }*	71.79%	15	*{ L.TI, L.lAmyg }*	67.87%
6	*{ L.vIa, L.lAmyg }*	71.79%	16	*{ L.TI, L.vIa, L.lAmyg }*	67.87%
7	*{ L.mAmyg, L.lAmyg }*	70.53%	17	*{ L.TI, L.vId/vIg }*	66.93%
8	*{ L.vIa, L.mAmyg }*	69.28%	18	*{ L.lAmyg, L.NAC }*	66.93%
9	*{ L.vId/vIg }*	68.50%	19	*{ L.TI, L.vIa, L.vId/vIg, L.lAmyg }*	66.93%
10	*{ L.vIa, L.vId/vIg }*	68.50%	20	*{ L.mAmyg, L.lAmyg, L.NAC }*	66.93%

**Figure 2:**
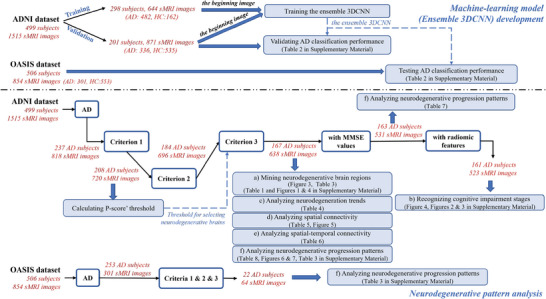
The data selection flowchart of this study. See the footnote of Table 2 for details on criteria 1, 2, and 3.

In the part of neurodegenerative pattern analysis, we further focus on AD subjects who meet the following three criteria in the ADNI database (including all samples in the training and validation datasets): 1) his/her longitudinal image sequence contains the images acquired at no less than two time points; 2) all sMRI images in his/her longitudinal image sequence are correctly classified by the *Ensemble 3DCNN*; 3) with the neuroimaging biomarker, *P*‐score, at least one degenerative brain region is identified in his/her longitudinal image sequence. In total, 167 AD subjects and their corresponding 638 longitudinal sMRI images are studied to explore the neuroimaging patterns of neurodegeneration in AD. After the neurodegenerative progression patterns are acquired with the ADNI database, the generalization of these patterns is also verified on the longitudinal image data in the OASIS database and the results are presented in the Supporting Information. **Figure** **2** illustrates the data selection flowchart in this study, and the detailed information of sMRI images involved in the six pattern analysis phases is shown in **Table** **2**.

In sum, 2369 (= 482+162+336+535+301+553) T1‐weighted structural MRI images of 1005 $=136+162+101+100+253+253) participants from ADNI and OASIS studies are applied to model derivation, validation, testing, and pattern analysis.

### 2.2 The DL‐Extracted Neuroimaging Biomarker *P*‐Score Facilitates Showing the Atrophy of the Classical Brain Regions Involved in AD

This section is corresponding to the two phases in the part of neurodegenerative pattern analysis: mining neurodegenerative brain regions and recognizing cognitive impairment stages, shown in Figure 1. Given an sMRI image, a neuroimaging biomarker named *P*‐score could be detected by mapping the AD predictive scores of the *Ensemble 3DCNN* to 246 fine‐grained regions in the Brainnetome Atlas.^[^ ^29^ ^]^

While *P*‐score acts as a regional biomarker associated with the probability of a brain region's involvement in AD progression, it effectively indicates the degree of neurodegeneration in each brain region.

**Figure 3:**
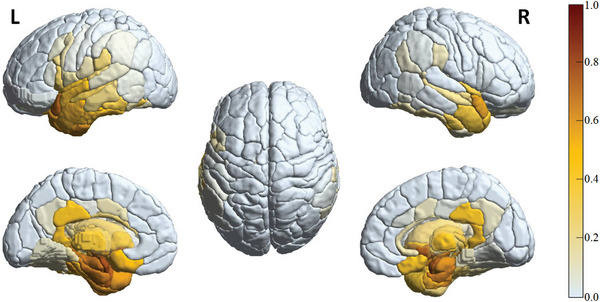
Illustration of the DL‐extracted neuroimaging biomarker (*P*‐score) in the 638 longitudinal sMRI images from the 167 AD subjects in the ADNI dataset.

For the convenience of observation, the *P*‐score value for each brain region is normalized to the range of [0,1]. We sum up *P*‐scores of all brain regions to get the whole‐brain *P*‐score, denoted as *P*‐score_whole_, to assess the degeneration of the entire brain. The higher *P*‐score value, the higher degree of neurodegeneration. In **Figure** **3**, we visually illustrate the average *P*‐score of each brain region in the 638 longitudinal sMRI images from the 167 AD subjects in the ADNI^[^ ^27^ ^]^ dataset. It is shown that the degree of neurodegeneration varies from region to region.

The*Ensemble 3DCNN* performs well in classifying sMRI images of AD subjects from those of HC, whose classification accuracy reaches 90% and 79% on the validation and testing datasets, respectively (see the Supporting Information for details). As shown in Table 2, the *Ensemble 3DCNN* correctly recognizes 720 of 818 AD sMRI images in the training and validation datasets from the ADNI database.^[^ ^27^ ^]^

We assume that the brain regions' *P*‐scores (after normalization) of AD patients follow a normal distribution, and design the threshold for neurodegeneration labeling based on the mean and standard deviation (std) of all brain regions' *P*‐score among all the AD sMRI images correctly identified by the *Ensemble 3DCNN* (i.e., 720 sMRI images from 208 AD subjects as shown in Table 2 and Figure 2). According to the statistics and comparisons of relevant trials, it is observed that a brain region is most likely not involved with the neurodegeneration associated with AD (Specificity = 0.99) if its *P*‐score is lower than λ = *mean* + 2 *std* = 0.73. Here, mean and std are the mean and the standard deviation of the *P*‐scores of all brain regions in the 720 AD sMRI images correctly identified by the *Ensemble 3DCNN*, respectively.

Therefore, we consider the brain regions with *P*‐score>λ as those involved with the neurodegeneration caused by AD, that is, the neurodegenerative brain regions. In the ADNI database,^[^ ^27^ ^]^ 208 AD subjects have sMRI images collected at no less than two time points, and 167 of the 208 AD subjects are correctly identified by the trained *Ensemble 3DCNN* and have at least one neurodegenerative brain region labeled by *P*‐score. An AD subject is considered to be correctly identified by the trained *Ensemble 3DCNN* if the model correctly classifies all the sMRI images in his/her longitudinal image sequence. Here, we select the 167 AD patients and the corresponding 638 MRI images for the subsequent pattern analysis experiments.

Our analytical results show that the left medial amygdala *(L.mAmyg)*, the left nucleus accumbens *(L.NAC)*, and the left lateral amygdala *(L.lAmyg)* are found to be the most common regions affected by AD. Here, the prefix capital letters L and R of a brain region label (e.g., *L.mAmyg*) refer to the left and the right cerebral hemisphere, respectively.

Specifically, the three brain regions are considered as neurodegenerative in *82.60*% *(527 sMRI images)*, *80.72*% *(515 sMRI images)*, and *73.20*% *(467 sMRI images)* of the 638 sMRI images labeled with AD (i.e., AD sMRI images), respectively. The common neurodegenerative brain regions, which frequently occur among the 167 AD subjects, are listed in **Table** **3**. Here, Apriori,^[^ ^33^ ^]^ a frequent item set mining algorithm, is applied to extract the common neurodegenerative brain regions in AD. The support rate is set as 0.5. The reported frequency corresponding to a brain region (/brain regions) in Table 3 means the probability that it is (/they are) neurodegenerative among the 638 sMRI images labeled with AD. Most of these common neurodegenerative brain regions are located in the basal portions of the putamen and accumbens nucleus. This result is consistent with amyloid deposits reported in Braak 1991.^[^ ^2^ ^]^ In addition, the neurodegenerative brain regions highly ranked by *P*‐score, such as the amygdala, nucleus accumbens, agranular insular cortex, and hippocampus, roughly match the isocortex, basal magnocellular complex, and transentorhinal regions identified in Braak 1991.^[^ ^2^ ^]^ It is observed that neurodegeneration often occurs in the left brain hemisphere. This might be associated with the fact that most subjects, that is, *89.45*% *(212 of 237)* AD subjects and *93.13*% *(244 of 262)* of the healthy controls, are right‐handed. See Table 1 for detailed information about the examined subjects. For more intuition, we also visualize the probability map of brain neurodegeneration in the AD population in the Supporting Information.

**Figure 4:**
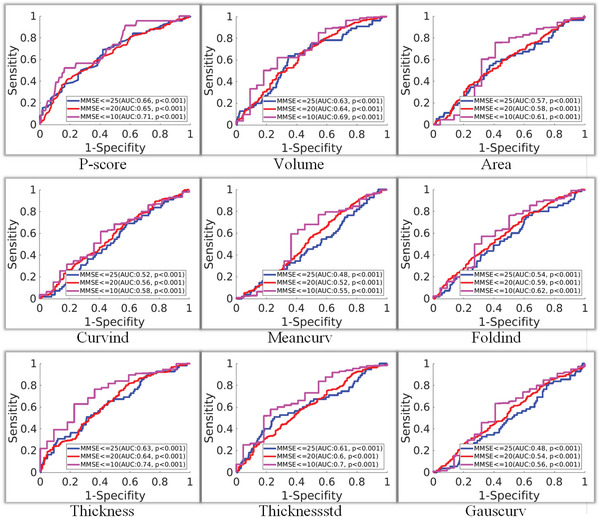
Receiver operating characteristic curves (ROC curves) in the three binary classification tasks on the recognition of different cognitive impairment levels indicated by MMSE using *P*‐score_whole_ and the existing radiomic features,^[^ ^34^ ^]^ respectively.

In addition to identifying the common neurodegenerative brain regions in AD progression, it is found that the *P*‐score_whole_, which is the sum of *P*‐score of all the brain regions, is more discriminative to recognize the cognitive impairment stage of AD subjects in comparison with the existing radiomic features.^[^ ^34^ ^]^ Here, eight radiomic features, *that is, gray matter volume in cortical layer (volume), surface area of cortical layer (area), intrinsic curvature index of cortical layer (curvind), rectified mean curvature of cortical layer (meancurv), folding index and intrinsic curvature index of cortical layer (foldind), average value of cortical thickness (thickness), standard deviation of cortical thickness (thicknessstd), integrated rectified Gaussian curvature of cortical layer (Gauscurv)*, are measured for sMRI images using the Surfer Software Suite (https://www.freesurfer.net/), which is an open‐source software suite for processing and analyzing (human) brain MRI images. To illustrate this point, we perform three binary classification tasks on recognizing different cognitive impairment levels indicated by Mini Mental State Examination (MMSE),^[^ ^35^ ^]^ that is, worse or better than the mild (cutoff MMSE=25), moderate (cutoff MMSE=20) or severe (cutoff MMSE=10) cognitive impairment stages, using *P*‐score_whole_. Here, 523 of 638 images of AD subjects are selected for the classification experiment, because the remaining 115 images have no corresponding MMSE values or radiomic feature value(s). The experimental results corresponding to the *P*‐score_whole_ and the existing radiomic features^[^ ^34^ ^]^ are shown in **Figure** **4**.

The first sub‐figure (i.e., at the top‐left corner) in Figure 4 illustrates the receiver operating characteristic curve (ROC curve) of recognizing the cognitive impairment stages using the *P*‐score_whole_ value for an AD subject in the three binary classification tasks. In the first sub‐figure, *P*‐score_whole_ exhibits a decent ability to identify severe cognitive impairment (Area under the ROC curve, i.e., AUC=71.0% with *p*<0.001 for MMSE ⩽10). The AD classification results of the *P*‐score are competitive to those of *Thickness* and *Thicknessstd*. In comparison with the *P*‐score_whole_, most existing radiomic features^[^ ^34^ ^]^ (except *Thickness* and *Thicknessstd*) are NOT good in directly recognizing the severe cognitive impairment level. Plus, although the obtained AUC results are relatively low when recognizing the mild or moderate cognitive impairment level with the *P*‐score_whole_, as shown in Figure 4, they are still better than those corresponding AUC results obtained with the radiomic features. The above validation experiments indicate that *P*‐score_whole_ could better predict the cognitive impairment status of AD subjects than most existing eight radiomic features.^[^ ^34^ ^]^ Moreover, the correlation between *P*‐score_whole_ and MMSE value is shown in the Supporting Information. For comparison, we also measure the eight radiomic features for sMRI images and plot the scatter diagram of these features and MMSE values in the Supporting Information.

### 2.3 The DL‐Extracted Neuroimaging Biomarker (*P*‐Score) Facilitates Showing the Progression Patterns of Neurodegeneration in AD as Defined Neuropathologically

This section is corresponding to the three phases in the part of neurodegenerative pattern analysis: analyzing neurodegeneration trends, spatial connectivity, and spatial‐temporal connectivity, shown in Figure 1. Advanced research^[^ ^36^, ^37^ ^]^ has indicated that AD tends to progress in a continuous and irreversible manner. Amyloid, *t*‐*tau*, NFTs, and other biochemical substances associated with nerve damage gradually accumulate in AD progression. The phenomena are also observed through the lens of the DL‐extracted neuroimaging biomarker (*P*‐score). We divide the value of *P*‐score at the region level into 20 grades (with a range of 0.05 for each degeneration grade) and investigate the fluctuations of *P*‐score for each brain region in the longitudinal sMRI images of an AD subject. As shown in Table 2, the 167 AD subjects with longitudinal sMRI images collected at multiple time points are enrolled from ADNI database for examination. Statistical results are summarized in **Table** **4**. In the neurodegenerative brain regions listed in Table 4, the grade of *P*‐score continues to rise (or hold unchanged) as the disease progresses for ≈*46.4*% of AD subjects on average. Around *33.7*% of AD subjects get increased *P*‐scores in the frequently labeled neurodegenerative brain regions over time with slight fluctuations. Here, an increase with slight fluctuations means that there is an acceptable decline (<0.1) in *P*‐score value over time, and the grade of *P*‐score at the last time point is higher than (or equal to) the one at the beginning. In summary, *P*‐scores of the common neurodegenerative brain regions listed in Table 4 increase over time in more than *80*% of AD subjects. In contrast, this deterioration trend of neurodegeneration can not be directly detected with the existing radiomic features.^[^ ^34^ ^]^

**Table 4:** Neurodegeneration trend in the common neurodegenerative brain regions

Brain region		Average *P*‐score among AD subjects	Percentage of AD patients with increased *P*‐score		
Abbreviation	Name		Continuously	With slight fluctuations	Total
*L.mAmyg*	Medial amygdala	0.798	42.5%	34.7%	77.2%
*L.NAC*	Nucleus accumbens	0.791	49.7%	33.5%	83.2%
*L.lAmyg*	Lateral amygdala	0.764	38.3%	30.0%	68.3%
*L.vIa*	Ventral agranular insula	0.760	38.3%	31.2%	69.5%
*L.TI*	Area TI	0.747	41.3%	30.0%	71.3%
*L.vId/vIg*	Ventral dysgranular and granular insula	0.745	37.1%	34.2%	71.3%
*R.mAmyg*	Medial amygdala	0.738	55.7%	30.5%	86.2%
*R.NAC*	Nucleus accumbens	0.735	50.3%	35.3%	85.6%
*L.A35/36r*	Rostral area 35/36	0.733	41.3%	34.1%	75.4%
*L.A38l*	Lateral area 38	0.727	38.3%	32.4%	70.7%
*L.rHipp*	Rostral hippocampus	0.694	50.9%	34.1%	85.0%
*L.A28/34*	Area 28/34 (EC)	0.677	55.1%	31.1%	86.2%
*R.A28/34*	Area 28/34 (EC)	0.671	57.5%	31.1%	88.6%
*R.lAmyg*	Lateral amygdala	0.670	45.5%	38.9%	84.4%
*L.vmPu*	Ventromedial putamen	0.655	47.3%	31.7%	79.0%
*R.vId/vIg*	Ventral dysgranular and granular insula	0.653	47.3%	36.5%	83.8%
*R.A35/36r*	Rostral area 35/36	0.650	43.1%	40.7%	83.8%
*R.vIa*	Ventral agranular insula	0.647	42.5%	39.5%	82.0%
*R.rHipp*	Rostral hippocampus	0.635	60.5%	31.1%	91.6%
Average		0.710	46.4%	33.7%	80.2%

**Figure 5:**
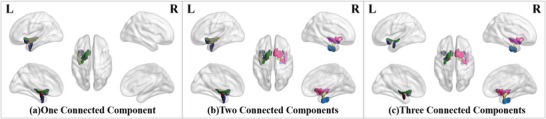
Different numbers of *spatially* connected components in the brain images of three different AD subjects. (Best view in color. Different regions are visualized with different colors.)

We consider that two brain regions are spatially adjacent if they respectively contain one of the two contiguous voxels, which share one common corner at least. It is observed that most neurodegenerative brain regions identified via *P*‐score are spatially interconnected. In only a few AD sMRI images, a small number of neurodegenerative brain regions are found to be sporadically isolated. To validate this observation, we apply the connected component analysis (CCA) algorithm (depth first search algorithm,^[^ ^38^ ^]^ i.e., DFS) to count the number of connected components in the neurodegenerative brain regions for each AD sMRI image. Details about the counting of connected components are introduced in the method section. As summarized in **Table** **5**, there are no more than three connected components in over *98*% *(631 of 638)* of AD sMRI images. Two connected components (one on each hemisphere of the brain in most cases) are detected in more than half of AD sMRI images, that is, around 55.5% *(354 of 638)*. As a visualization example, sMRI images with different numbers of connected components are mapped onto the brain atlas in **Figure** **5**. Based on the results of spatial connectivity analysis in Table 5, the neurodegenerative brain regions caused by AD are more likely to be spatially connected at any time in AD progression.

**Table 5:** Results of *spatial* connected component analysis for the neurodegenerative brain regions in AD sMRI images

Model	No. of connected components	Left brain hemisphere		Right brain hemisphere		Whole brain	
		No. of images	Percentage	No. of images	Percentage	No. of images	Percentage
	=0	116	18.18%	255	39.97%	89	15.56%
	⩽1	544	85.27%	623	97.65%	231	36.21%
*Ensemble*	⩽2	635	99.53%	638	100%	585	91.69%
*3DCNN*	⩽3	638	100 %	–	–	631	98.90%
	⩽4	–	–	–	–	637	99.84%
	⩽5	–	–	–	–	638	100 %

The spatial‐temporal connectivity of the neurodegenerative brain regions affected by AD has also been examined in the longitudinal sMRI images of AD subjects. Here, in the longitudinally adjacent MRI images of an AD subject, the same brain regions are considered spatio‐temporal adjacent. We detect at least one neurodegenerative region in the longitudinal sMRI images of each of the 167 AD subjects using *P*‐score. Since we set λ with a strict threshold to ensure the reliability in detecting neurodegenerative regions, no neurodegenerative brain region is detected via *P*‐score in the 17 of 184 AD subjects, as shown in Table 2. Similar to the spatial connectivity analysis in an sMRI image at a single time point, the CCA algorithm is applied to examine the spatial‐temporal connectivity of neurodegenerative brain regions detected in the longitudinal sMRI images. Results of spatial‐temporal CCA for the neurodegenerative brain regions caused by AD are summarized in **Table** **6**. Compared with the results in Table 5, no significant shift is detected in the percentages located in each range of the number of connected components. This result means that the number of connected components does not sharply increase even if images taken at different time points in a longitudinal sMRI sequence are added into the analysis. In other words, the connectivity of the neurodegenerative brain regions acquired at a single time point holds up to longitudinal (multiple time points) analysis during AD progression.

**Table 6:** Results of *spatial‐temporal* connected component analysis for the neurodegenerative brain regions in *longitudinal* AD sMRI images

Model	No. of connected components	Left brain hemisphere		Right brain hemisphere		Whole brain	
		No. of patients	Percentage	No. of patients	Percentage	No. of patients	Percentage
	=0	9	5.39%	34	20.36%	0	0.0%
*Ensemble*	⩽1	136	82.04%	159	95.21%	29	17.37%
*3DCNN*	⩽2	166	99.40%	167	100%	144	86.23%
	⩽3	167	100%	–	–	165	98.80%
	⩽4	–	–	–	–	167	100%

### 2.4 The DL‐Extracted Neuroimaging Biomarker (*P*‐Score) Facilitates Identifying the Involvement Sequence of Neurodegenerative Brain Regions Affected by AD Over Time

This section is corresponding to the sixth phase in the part of neurodegenerative pattern analysis, that is, analyzing neurodegenerative progression patterns, shown in Figure 1. In this section, first, the 638 images from the 167 AD patients are divided into different stages of AD according to their corresponding MMSE values. Second, the proportion of common neurodegenerative brain areas was analyzed for the images corresponding to different stages of the disease. The aim is to answer the question that whether the neurodegenerative percentage of each brain region in the 638 images varies from stage to stage and what the patterns are. Third, for these 167 AD patients, the first image at each stage of AD is selected for analysis to see if the neurodegenerative proportion of each brain region in the 167 AD patients varies across stages. Fourth, we analyze the longitudinal imaging data of each of the 167 AD patients to observe the spatial relationship between the newly neurodegenerative brain region(s) and the previously neurodegenerative brain region(s) for each AD patient as the disease progresses. Finally, we investigate whether common sequential patterns of neurodegenerative brain regions could be observed in these 167 AD patients. And if so, we further acquire them. In addition, to test the acquired sequential patterns from the ADNI database, we employ the longitudinal image data in the OASIS cohort to test the obtained patterns, and present the testing results in the Supporting Information.

As shown in Figure 2, the 531 sMRI images of the 163 AD subjects are divided into several groups according to their cognitive impairment level (MMSE^[^ ^35^ ^]^ value). Here, the 531 of the 638 sMRI images of AD subjects are selected for the experiment, because the remaining 107 images have no corresponding MMSE values. We explore the frequent item‐sets consisting of the neurodegenerative regions in each level (normal: MMSE ∈ [27, 30]; mild: MMSE ∈ [21, 26]; moderate: MMSE ∈ [10, 20]; severe: MMSE ∈ [0, 9]) to analyze the process of neurodegeneration at different stages of AD. As summarized in **Table** **7**, in the early stage of cognitive impairment (MMSE ∈ [27, 30]), *L.mAmyg* and *L.NAC* have been neurodegenerative in most *(over 83%)* AD sMRI images. Moreover, in more than *55%* of AD sMRI images in the early cognitive impairment stage, the degeneration is found in the *L.lAmyg*, *L.TI*, *L.vIa*, *L.A35/36r*, and *R.mAmyg*. Besides, the *L.vId/vIg*, the *A38l* and *R.NAC* are degenerative in over *50%* of AD sMRI images in the early stage of cognitive impairment (MMSE ∈ [27, 30]). As the cognitive impairment further worsens (MMSE < 20), *L.rHipp* is increasingly involved among the 638 sMRI images of the 167 AD subjects. In general, the frequency of the neurodegenerative brain regions *(such as L.lAmyg, L.vIa, L.vId/vIg, L.A38l, R.NAC, L.rHipp, L.A28/34, and R.A28/34)* rises with increasing cognitive impairment.

**Table 7:** Frequency of the neurodegenerative brain regions in all *MMSE* levels

Degenerative region	Cognitive impairment level (MMSE [35])			
	Normal ([27, 30])	Mild ([21, 26])	Moderate ([10, 20])	Serve ([0, 9])
*L.mAmyg*	83.3%	74.8%	79.4%	86.7%
*L.NAC*	83.3%	73.6%	80.0%	93.3%
*L.lAmyg*	61.1%	63.2%	70.6%	80.0%
*L.TI*	61.1%	58.5%	65.6%	73.3%
*L.vIa*	55.6%	62.3%	69.4%	80.0%
*L.A35/36r*	55.6%	53.5%	62.8%	73.3%
*R.mAmyg*	55.6%	53.1%	65.6%	80.0%
*L.vId/vIg*	50.0%	58.8%	67.2%	80.0%
*L.A38l*	50.0%	54.4%	62.2%	66.7%
*R.NAC*	50.0%	51.6%	60.6%	80.0%
*L.rHipp*	27.8%	37.4%	47.2%	66.7%
*L.A28/34*	16.8%	23.0%	29.4%	46.7%
*R.A28/34*	11.1%	18.9%	21.7%	40.0%

Note: Colors red, blue, green, and dark are utilized to indicate the data in the range [80%, 100%], [60%, 80%), [40%, 60%), and [0%, 40%), respectively.

We further analyze the involvement sequence of neurodegenerative brain regions in the 638 longitudinal sMRI images of the 167 AD subjects shown in Table 2.

According to the experimental results, it is found that *L.mAmyg*, *L.NAC*, and *L.lAmyg* are the most common neurodegenerative regions in the early stage of AD subjects, accounting for *68.26*% *(114)*, *65.87*% *(110)*, and *58.08*% *(97)* of the 167 AD subjects, respectively. As time goes on, more brain regions could be involved in degeneration in *90.42*% *(151)* of the 167 AD subjects.

Moreover, it is interesting to notice that the newly added neurodegenerative region or regions are likely to be around the previous one(s). This may be the major reason for no significant change between the results of spatial and spatial‐temporal connectivity analysis, as exhibited in Tables 5 and 6, respectively. More specifically, we notice that the number of connected components increases over time in *78 (out of 167)* AD subjects. In *15* out of the rest *89* AD subjects, the number of connected components is reduced because the newly involved neurodegenerative region or regions make those isolated previously get connected. The remaining *74* AD subjects keep the number of connected components unchanged, as the newly added neurodegenerative regions are around those previously existing.

Furthermore, we observe that neurodegeneration in caudal or (dorso)lateral regions commonly occurs after the atrophy in the basal area. To verify this observation, we obtain the frequent sequential patterns of items (i.e., the neurodegenerative regions), in the longitudinal AD sMRI images. Here, Sequential PAttern Discovery using Equivalence classes (SPADE) algorithm^[^ ^39^ ^]^ is applied for mining frequent sequential patterns.

**Figure 6:**
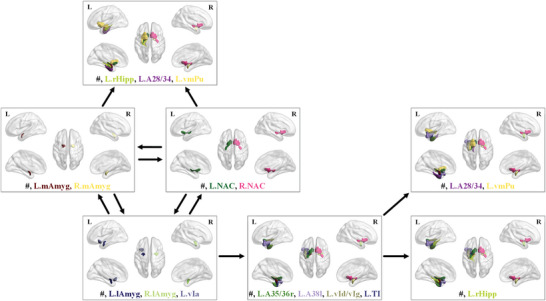
Illustration of multiple progression trajectories of structural neurodegeneration across brain regions discovered via *P*‐score. (Best view in color. Different regions are visualized in different colors, corresponding to the colors of their name abbreviations written at the bottom of each sub‐figure. Symbol # represents the elements in the antecedent.)

**Figure 7:**
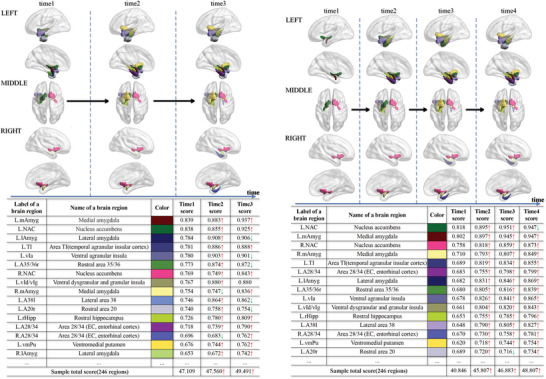
Examples of neurodegenerative sequences obtained via *P*‐score for two AD subjects. (Best view in color. Different regions are visualized with different colors. Due to page limit, *P*‐score of the neurodegenerative brain regions is partially listed in the subfigures.)

The most common sequential patterns are partially listed in **Table** **8**. That is, Table 8 lists neurodegenerative sequences with a supporting rate greater than *18%*. More common neurodegenerative sequences and other relevant results can be found in the Supporting Information. According to this result, no sequential pattern with a supporting rate greater than *29%* is acquired, which indicates that heterogeneous neurodegenerative sequences of brain regions might be involved in AD progression. Even so, multiple progression trajectories of neurodegeneration can be observed. 1) For the same brain region in both hemispheres, the left side of the brain commonly degenerates before the right. For instance, neurodegenerative sequences of *{L.NAC}* ⟶ *{#, R.NAC}* and *{L.mAmyg}* ⟶ *{#, R.mAmyg}* are detected. 2) The brain neurodegeneration of AD subjects can start in *NAC* and spread to *mAmyg* or vice versa. 3) *rHipp)* and/or *A28/34*, and/or *vmPu* may be atrophy after the degeneration of *NAC* and/or *‐mAmyg*. 4) vIa and/or *lAmyg* may join the trajectory cycle of *NAC* and *mAmyg*. 5) *lAmyg* and/or vIa may shrink before the atrophy of *A38l*, and/or *TI*, and/or *A35/36r*, and/or *vId/vIg*, followed by the degeneration of *rHipp*. The shrinkage of entorhinal cortex *(A28/34)* and/or *vmPu* may also appear after the degeneration of t *A38l*, and/or *TI*, and/or *A35/36r*, and/or *vId/vIg*. More intuitively, these discovered multiple progression trajectories of structural neurodegeneration across brain regions are demonstrated in **Figure** **6**. Also, we illustrate the neurodegenerative sequences of two AD subjects as examples in **Figure** **7**.

**Table 8:** Frequent sequential patterns of the neurodegenerative brain regions in the longitudinal sMRI image sequences of AD subjects

No.	Frequent sequential patterns	Frequency
1	*{L.NAC }* ⟶ *{ #, L.A28/34}*	28.1%
2	*{L.mAmyg, L.NAC}* ⟶ *{ #, L.A28/34}*	27.0%
3	*{L.NAC}* ⟶ *{ #, R.A28/34}*	24.0%
4	*{L.vIa, L.lAmyg}* ⟶ *{ #, L.vmPu}*	23.4%
5	*{ L.mAmyg }* ⟶ *{ #, L.vmPu}*	23.4%
6	*{L.mAmyg}* ⟶ *{ #, L.rHipp}*	23.4%
7	*{L.vIa, L.mAmyg, L.lAmyg, L.NAC}* ⟶ *{ #, L.A28/34}*	23.4%
8	*{L.mAmyg, L.NAC}* ⟶ *{ #, L.vmPu}*	22.8%
9	*{R.NAC}* ⟶ *{ #, R.A28/34}*	22.8%
10	*{L.NAC}* ⟶ *{ #, L.rHipp}*	22.8%
11	*{L.NAC}* ⟶ *{ #, R.mAmyg}*	22.8%
12	*{R.mAmyg}* ⟶ *{ #, R.A28/34}*	22.8%
13	*{L.mAmyg, L.NAC}* ⟶ *{ #, R.A28/34}*	22.8%
14	*{L.vIa, L.mAmyg, L.lAmyg }* ⟶ *{ #,L.vmPu}*	22.8%
15	*{L.A38l, L.TI, L.vIa, L.vId/vIg, L.mAmyg, L.lAmyg}* ⟶ *{ #, L.vmPu}*	22.2%
16	*{L.NAC}* ⟶ *{ #, R.NAC}*	22.2%
17	*{L.NAC, R.NAC}* ⟶ *{ #, R.A28/34}*	22.2%
18	*{L.vIa, L.mAmyg, L.lAmyg, L.NAC}* ⟶ *{ #, L.vmPu}*	22.2%
19	*{R.mAmyg, R.NAC}* ⟶ *{ #, R.A28/34}*	22.2%
20	*{L.A38l, L.A35/36r, L.TI, L.vIa, L.vId/vIg, L.mAmyg, L.lAmyg}* ⟶ *{ #, L.vmPu}*	21.6%
21	*{L.mAmyg, R.mAmyg, L.NAC}* ⟶ *{ #, L.A28/34}*	21.6%
22	*{R.mAmyg, L.NAC, R.NAC}* ⟶ *{ #, R.A28/34}*	21.6%
23	*{L.mAmyg, L.NAC, R.NAC}* ⟶ *{ #, R.A28/34}*	21.6%
24	*{L.mAmyg, L.NAC}* ⟶ *{ #, L.rHipp}*	21.6%
25	*{L.TI, L.vIa, L.vId/vIg, L.mAmyg, L.lAmyg, L.NAC}* ⟶ *{ #, L.A28/34}*	21.6%
26	*{L.A38l, L.TI, L.vIa, L.vId/vIg, L.mAmyg, L.lAmyg, L.NAC}* ⟶ *{ #, L.vmPu}*	21.6%
27	*{L.mAmyg}* ⟶ *{ #, R.mAmyg}*	21.0%
28	*{L.mAmyg, R.mAmyg, L.NAC, R.NAC}* ⟶ *{ #, R.A28/34}*	21.0%
29	*{L.A38l, L.A35/36r, L.TI, L.vIa, L.vId/vIg, L.mAmyg, L.lAmyg, L.NAC}* ⟶ *{ #, L.vmPu}*	21.0%
30	*{L.mAmyg}* ⟶ *{ #, R.NAC}*	20.4%
31	*{L.vIa, L.lAmyg}* ⟶ *{ #, L.rHipp}*	20.4%
32	*{L.mAmyg, L.NAC}* ⟶ *{ #, R.mAmyg}*	20.4%
33	*{L.A35/36r, L.TI, L.vIa, L.vId/vIg, L.mAmyg, L.lAmyg, L.NAC}* ⟶ *{ #, L.A28/34}*	20.4%
34	*{L.A38l, L.TI, L.vIa, L.vId/vIg, L.mAmyg, L.lAmyg, L.NAC}* ⟶ *{ #, L.A28/34}*	20.4%
35	*{L.mAmyg, L.NAC, R.NAC}* ⟶ *{ #, L.A28/34}*	19.8%
36	*{L.vIa, L.mAmyg, R.mAmyg, L.lAmyg, L.NAC}* ⟶ *{ #, L.A28/34}*	19.8%
37	*{L.mAmyg, L.NAC}* ⟶ *{ #, R.NAC}*	19.8%
38	*{L.A38l, L.A35/36r, L.TI, L.vIa, L.vId/vIg, L.mAmyg, L.lAmyg, L.NAC}* ⟶ *{ #, L.A28/34}*	19.8%
39	*{L.A38l, L.A35/36r, L.TI, L.vIa, L.vId/vIg, L.mAmyg, L.lAmyg, L.rHipp, L.NAC}* ⟶ *{ #, L.vmPu}*	19.2%
40	*{L.mAmyg, R.mAmyg, L.NAC}* ⟶ *{ #, L.vmPu}*	19.2%
41	*{L.TI, L.vIa, L.lAmyg}* ⟶ *{ #, L.rHipp}*	19.2%
42	*{L.vIa, L.mAmyg, L.lAmyg}* ⟶ *{ #, L.rHipp}*	19.2%
43	*{L.vIa, L.mAmyg, R.mAmyg, L.lAmyg, L.NAC}* ⟶ *{ #, L.vmPu}*	18.6%
44	*{L.TI, L.vIa, L.mAmyg, L.lAmyg}* ⟶ *{ #, L.rHipp}*	18.6%
45	*{L.TI, L.vIa, L.vId/vIg, L.lAmyg}* ⟶ *{ #, L.rHipp}*	18.0%
46	*{L.mAmyg}* ⟶ *{ #, R.lAmyg}*	18.0%
47	*{L.vIa, L.lAmyg}* ⟶ *{ #, R.A28/34}*	18.0%
48	*{L.mAmyg, R.mAmyg, L.NAC, R.NAC}* ⟶ *{ #, L.A28/34}*	18.0%
49	*{L.TI, L.vIa, L.vId/vIg, L.mAmyg, R.mAmyg, L.lAmyg, L.NAC}* ⟶ *{ #, L.A28/34}*	18.0%
50	*{L.A38l, L.TI, L.vIa, L.vId/vIg, L.mAmyg, R.mAmyg, L.lAmyg, L.NAC}* ⟶ *{ #, L.vmPu}*	18.0%

Note: Symbol # represents the elements in the antecedent.

## 3 Discussion

### 3.1 Summary of the Main Findings

In this literature, a new neuroimaging biomarker named *P*‐score is devised on the basis of the AD predictive scores from the *Ensemble 3DCNN*. Our results show the effectiveness of *P*‐score in identifying classical neurodegenerative brain regions, such as the medial amygdala, nucleus accumbens, lateral amygdala, during AD progression. Good capability in recognizing the subjects with less severe cognitive impairment is achieved with the help of *P*‐score. Furthermore, together with DFS^[^ ^38^ ^]^ and SPADE^[^ ^39^ ^]^ algorithms, *P*‐score is utilized to help verify the spatial/spatial‐temporal connectivity and increased involvement of the neurodegenerative brain regions in AD progression. Although the obtained involvement pattern seems to be heterogeneous in longitudinal AD sMRI images, multiple longitudinal trajectories of involvement of neurodegenerative brain regions associated with AD are detected via *P*‐score.

### 3.2 Comparison to the Previous Work

Similar observations in terms of spatial‐temporal connectivity and continuity of neurodegenerative brain regions related to AD are obtained via *P*‐score, in comparison with those acquired via biochemical markers *(such as amyloid, t‐tau, NFTs, and neuropil threads (NTs))*. Many neurodegenerative brain regions detected with *P*‐score are located within or near areas highlighted in Braak 1991.^[^ ^2^ ^]^ *P*‐score can facilitate revealing additional neurodegenerative patterns in comparison with Braak 1991.^[^ ^2^ ^]^

1) The area of *NAC* corresponds to the basal magnocellular complex, where NFTs/NTs are observed sparsely and substantially after Stages II and IV, respectively. In fact, a growing body of research has recently indicated apathy is one of the earliest signs of dementia, for example, the previous work^[^ ^40^ ^]^ provides novel evidence for apathy as a prodrome of dementia. Just as Guo 2022^[^ ^41^ ^]^ mentioned, it is this neuronal damage in the nucleus accumbens that likely causes the apathy and motivational problems that can signal the earliest stage of Alzheimer's disease. This is consistent with the neurodegenerative sequence of NAC shown in Figure 6 to a great extent.
2) Area 28/34 *(A28/34)* and TI are entangled with the parahippocampal gyrus (such as the subiculum, entorhinal, and transentorhinal areas). Although these regions and the basal magnocellular complex are commonly filled with a large number of NFTs and NTs in the brain of AD subjects according to Braak 1991,^[^ ^2^ ^]^ obvious neurodegenerative sequence between the parahippocampal gyrus and the basal magnocellular complex was NOT explicitly reported in Braak 1991.^[^ ^2^ ^]^ Here, with the examination of *P*‐score among sMRI images of AD subjects, the parahippocampal gyrus *(A28/34)* is found to neurologically degenerate after the morphological change in the basal magnocellular complex (NAC), which is partially consistent with a shift in the distribution of temporal lobe atrophy with advancing disease.^[^ ^42^, ^43^ ^]^
3) In the olfactory pathway, as the only sensory pathway that does not relay in the thalamus, axons from mitral cells in the olfactory bulb form the olfactory tract and synapse in various cortical regions, for example, anterior olfactory nucleus, piriform cortex, the medial amygdala *(mAmyg)* and entorhinal cortex *(A28/34)*. Especially, the medial amygdala *(mAmyg)* is a central hub in the olfactory neural network. The brain regions of mAmyg and *A28/34* are involved in AD progression, which is consistent with the invariable and severe involvement of the olfactory areas of the brain in AD, just as indicated in Pearson 1985.^[^ ^44^ ^]^ Here, the spread from medial amygdala to the parahippocampal gyrus is explicitly disclosed in Figure 6. In addition, the Amygdala and hippocampus *(lAmyg, rHipp)* are the brain regions associated with the striate area, which is the visual field characterized by amyloid depositions. Although Braak et al.^[^ ^2^ ^]^ observed NFTs/NTs accumulate in the striate area in Stages V–VI of AD, they did not explicitly indicate the neurodegenerative sequence between the amygdala and hippocampus. In contrast, with *P*‐score, we observe that the amygdala *(mAmyg, lAmyg)* commonly changes in morphology before the hippocampus *(rHipp)* does during AD progression, as shown in Figure 6.
4) There remains a slight bias between the neurodegenerative areas detected via *P*‐score and those identified with biochemical markers, for example, NFTs and NTs. Braak et al.^[^ ^2^ ^]^ suggested that the insula area *(vIa, vIg)* was less relevant to AD progression, but this region is marked by *P*‐score as a common neurodegenerative region in early and advanced AD. However, the previous researches^[^ ^45^, ^46^, ^47^, ^48^, ^49^, ^50^ ^]^ indicated that the insula may be involved early in Alzheimer's disease from the perspective of insular gray matter (GM) loss, abnormal insular activities and disrupted insular network etc., and that atrophy of the insular cortex may contribute to the cognitive deficits typical of early Alzheimer's disease. This is consistent with the phenomena exhibited in Figure 6.
5) The observation that the newly added neurodegenerative region or regions are likely to be around the previous one(s) supports the suggestion in the studies,^[^ ^43^, ^44^ ^]^ that is, the pathological changes in Alzheimer's disease affect regions that are interconnected by well‐defined groups of connections and that the disease process may extend along the connecting fibers, to a great degree.

The differences between observations with *P*‐score and those in Braak 1991^[^ ^2^ ^]^ could be due to individual differences in the subjects enrolled in the different trials. Another reason might be that biochemical markers focus on areas where interneuronal transmission mediators diffuse, while the examination with *P*‐score identifies brain regions where morphological changes are significant. After all, the DL‐extracted neuroimaging biomarker (i.e., *P*‐score) and the biochemical markers focus on structural/morphological and neuropathological changes of the brain, respectively. Meanwhile, since the structural changes in the AD brain are associated with the AD neurodegenerative progression, the progression patterns identified with biochemical markers are consistent with those disclosed with the DL‐extracted neuroimaging biomarker to some extent. In addition, as AD subjects enrolled in the ADNI database^[^ ^27^ ^]^ are more likely to be in the early or middle stages, the patterns of AD progression detected via *P*‐score are more in line with those in the middle stages (Stages II–IV) of Braak 1991.^[^ ^2^ ^]^ As illustrated in Figure 6, the neurodegeneration of NAC *(basal magnocellular complex, where NFTs and NTs present after Stage I)* occurs before that of *A28/34* *(subiculum, where NFTs and NTs are detected after Stage III)* and that of rHipp *(striate area, where NFTs and NTs present after Stage III)*.

In addition, this study is different from the previous work^[^ ^51^, ^52^, ^53^, ^54^, ^55^ ^]^ in the method and experimental results. Specifically, the method mentioned in Qiu 2020^[^ ^51^ ^]^ uses a deep learning model (fully convolutional neural network, FCNN) to learn volumetric patches obtained by random sampling in the brain to obtain disease probability maps; and then, from the maps, the voxels of high‐risk were selected to train a multilayer perceptron (MLP) for binary classification of disease states, that is, AD versus HC; meanwhile, an MLP model was trained to classify AD and NC using non‐neuroimaging features, including age, gender, and MMSE; finally, these two MLP models were fused for binary classification of AD and NC. Although this method of obtaining the disease probability map is somewhat similar to one or two steps in the *P*‐score calculation, the difference is very obvious. First, the two methods have different focuses. The method proposed in Qiu 2020^[^ ^51^ ^]^ mainly focuses on using the voxels of high‐risk to improve the effectiveness of binary classification for individuals based on the MRI images at a single time point; the *P*‐score proposed here mainly focuses on a probabilistic score regarding membership of the given input sMRI image to the AD class as the output of the trained binary classification model, that is, *Ensemble 3DCNN*, while an MRI image is input. Second, the two methods have different purposes. The purpose of proposing the disease probability map in Qiu 2020^[^ ^51^ ^]^ is to be able to perform binary classification with higher accuracy and better interpretability for individuals using the MRI images at a single time point while the purpose of advocating the *P*‐score is to detect the neurodegenerative patterns of AD progression through the eyes of neuroimaging. Finally, the research scope and results accomplished with the two methods are different. Qiu 2020^[^ ^51^ ^]^ focuses on the image‐feature MLP model based on the disease probability map and its fusion model combined with the non‐image feature MLP model to obtain an interpretable DL framework with satisfactory prediction performance verified by multicentre experiments and practicing neurologists. In contrast, this paper focuses on, with the help of *P*‐score derived from *Ensemble 3DCNN*, investigating the neurodegenerative brain regions in AD; identifying the cognitive impairment stage; recognizing the time‐varying, space‐varying and spacetime‐varying neurodegenerative patterns associated with the identified neurodegenerative brain regions (i.e., approximate monotonicity and connectivity) when only space, only time, and neither space nor time are fixed in longitudinal neuroimaging studies, respectively; acquiring and testing multiple progression trajectories of structural neurodegeneration across brain regions, followed by comparing them with the reported patterns in Braak 1991^[^ ^2^ ^]^; and at last, quite interesting and promising experimental results are obtained.

In Feng 2022,^[^ ^52^ ^]^ the “deep learning MRI” score (DLMRI), derived from the deep learning model trained on AD dementia, is proposed to detect prodromal AD and to predict time to dementia progression. Meantime, a 3D class activation map is generated to evaluate the regional contribution to AD classification. DLMRI outperforms other neuroimaging biomarkers of neurodegeneration in prodromal AD and the biomarkers of amyloid and tau pathology, which can support the *P*‐score advanced here as a technical basis because both Feng 2022^[^ ^52^ ^]^ and this study suggest that the continuous output from the trained DL classification model is reflective of the progressive structural patterns of AD pathology. However, unlike *P*‐score, DLMRI is unable to directly help reveal the time‐varying, space‐varying and

spacetime‐varying neurodegenerative patterns across brain regions in AD progression.

In Giorgio 2022,^[^ ^53^ ^]^ a robust and interpretable machine learning approach is utilized to quantify interactions between key pathological markers (β‐amyloid, medial temporal lobe atrophy, *tau* and APOE 4) at mildly impaired and asymptomatic stages of AD. In Giorgio 2020,^[^ ^54^ ^]^ a novel trajectory modeling approach based on metric learning (i.e., generalized metric learning vector quantization) is advocated to mine multimodal data from MCI patients in the ADNI cohort to derive individualized prognostic scores of cognitive declines due to AD. Yet, neither of these two approaches can help disclose neurodegenerative patterns associated with brain regions involved in disease progression.

In Popuri 2020,^[^ ^55^ ^]^ the ensemble‐learning framework that combines structural features in most discriminative ROIs is utilized to create an aggregate measure of neurodegeneration in the AD brain. Thus, as the output of the classifier ensemble and a continuous scalar score, between [0–1], an MRI‐based dementia of Alzheimer's type (DAT) score (MRDATS for short) is advocated to mimic the continuous influence of the AD pathology in the alterations observed in brain MR structural patterns. Although it is possible for the score to be directly employed to quantify the neurodegeneration inherent in the structural patterns in the 3D MRI image of the individual, Popuri 2020^[^ ^55^ ^]^ does NOT present the acquired structural patterns or quantified neurodegeneration in them with the help of MRDATS. Meantime, the proposed ensemble‐learning framework has NOT taken advantage of the powerful capability of deep learning in feature extraction and representation to generate the MRDATS.

Plus, as a matter of fact, the heterogeneity in AD,^[^ ^56^ ^]^ that is, variations in its clinical manifestations and biomarker longitudinal trajectories, has been investigated, and several potential subtypes have been identified in the recent studies.^[^ ^57^, ^58^, ^59^, ^60^, ^61^, ^62^, ^63^ ^]^ Most studies employ unsupervised clustering methods,^[^ ^57^, ^58^, ^59^ ^]^ and some leverage semi‐supervised clustering methods.^[^ ^59^, ^60^, ^61^, ^64^ ^]^ Since the datasets and methods used are different and the number of clusters together with the anatomical patterns of subtypes vary across studies, the results of these studies cannot be directly compared. Anyway, the results of our study also further reveal the heterogeneity of AD in the neurodegenerative sequence of brain regions to some extent. The next step might be to validate the proposed methods and the obtained patterns here in a larger AD population (together with other modalities including PET and/or single nucleotide polymorphisms) and to investigate the heterogeneity exhibited by the acquired patterns.

In sum, as an DL‐extracted neuroimaging biomarker, *P*‐score offers several additional advantages over existing biomarkers: 1) *P*‐score facilitates observing morphological changes in neurodegeneration at a fine‐grained level on the basis of sMRI images. According to the Brainnetome Atlas,^[^ ^29^ ^]^ the whole brain is partitioned into 246 regions. *P*‐score can be helpful to elaborately assess the degree of neurodegeneration in each brain region and detect the neurodegenerative sequence among these small regions. 2) *P*‐score can act as a quantitative indicator that facilitates evaluating the probability of an event that a brain (region) is impacted by AD‐related neurodegeneration through the eyes of neuroimaging. More importantly, *P*‐score values are comparable across different brain regions and/or across different individuals. 3) It is relatively intuitive and clinically easy to utilize *P*‐score to fine‐grainedly track and predict AD progression in individuals and the effects of treatment on them since it is calculated on the basis of non‐invasive sMRI images collected in vivo. Hopefully, with the help of the proposed *P*‐score here, AD patients will undergo an individualized staging evaluation on the basis of an expert‐consensus benchmark cohort built in an interactive way between cohort and model with interdisciplinary efforts to improve individualized diagnosis and therapeutic intervention strategies.

### 3.3 Limitations

As *Ensemble 3DCNN* is a data‐driven DL approach, the effectiveness in analysis of neurodegenerative progression using *P*‐score is affected by the bias in data collection. Most subjects enrolled in the ADNI study^[^ ^27^ ^]^ could suffer from AD for some time before their first examination saved in the ADNI database. Therefore, our method could hardly explore the neurodegeneration in the initial stage of AD in terms of neuroimaging. Similarly, due to the limited number of AD subjects available, the ability of *P*‐score to recognize neurodegeneration in the terminal stage of AD has not been fully verified. Moreover, longitudinal sMRI images of most subjects are collected at about 3–6 time points, resulting in the inability of our experiments to explore long‐term neurodegenerative patterns in AD ranging from initial to terminal stages. The relatively small number of time points for data collection also leads to the limited capability of the *Ensemble 3DCNN* model to mine the underlying sequential patterns of neurodegeneration in AD progression using sMRI images.

### 3.4 Future Development and Direction

On the one hand, the clinical implication and heterogeneity of the finding together with the generalizability of the advocated *P*‐score and method need to be further examined in a larger AD population in future research. The proposed method may be useful for in vivo staging of AD based on other image modalities and multi‐modality imaging, such as PET and other MRI modality, for example, T2‐weighted‐fluid‐attenuated inversion recovery (T2‐FLAIR), proton‐density weighted (PD), in a comprehensive manner. Moreover, the interactions and associations among brain regions during neurodegeneration of AD are other valuable topics, which we will further investigate to stimulate the in vivo staging of AD. On the other hand, we will further enhance the quantitative indicators derived from the state‐of‐the‐art explainable deep learning models for fine‐grained measuring the progress of the disease based on neuroimaging in a more sophisticated and interpretable way. The time bias problem in data collection warrants alleviation and the sequence alignment method, such as dynamic time wrapping, needs to be investigated. Data mining algorithms using limited data will also be tested in *P*‐score sequences to explore more refined neurodegenerative patterns in AD progression.

### 3.5 Contributions and Significance of the Work

*Neurodegenerative trajectories*: This study investigates the feasibility of DL in facilitating verifying the neurodegenerative changes in AD progression. We reexamine human neurodegeneration from the standpoint of neuroimaging and artificial intelligence, which allows automatic exploration of trajectory patterns during AD progression. Our method benefits from the powerful representation capabilities of deep learning, enabling us to examine subtle structural changes in the brain during AD using sMRI images. The neurodegeneration patterns are detected at a fine‐grained level by analyzing the neurodegenerative sequences of hundreds of brain regions. Our study helps stimulate the research on fine‐grained tracking and prediction of AD progression in individuals and the effects of treatments on them from the perspective of the analysis of non‐invasive in vivo neuroimaging data.

*Clinical impacts*: We derive a new neuroimaging biomarker (*P*‐score) from the *Ensemble 3DCNN* model to detect critical neurodegenerative changes in AD using sMRI. As a non‐invasive in vivo examination process based on sMRI images, our method helps identify and evaluate the various stages of AD. The disease progression patterns detected via *P*‐score might be applied to predict the next neurodegenerative brain region(s), neurodegenerative course, and the associated impaired functions (e.g., clinical symptoms) for individual AD subjects. Furthermore, it is with the help of *P*‐score that the easily available and repeatable sMRI examination could provide more subtle and rigorous monitoring for AD progression, which is valuable for tracking disease progression in individuals and the effects of treatment. In summary, this study offers effective means and new scientific evidence derived from DL models to detect fine‐grained neurodegenerative changes in the AD brain through the lens of neuroimaging.

## 4 Experimental Section

### Deep Learning Model *(Ensemble 3DCNN)* Development

**Figure 8:**
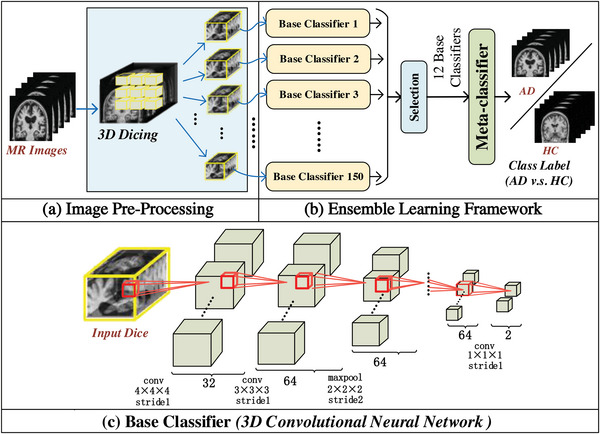
Illustration of the *Ensemble 3DCNN*. a) Image pre‐processing; b) Ensemble learning framework; c) Network architecture of each base classifier *(3DCNN)*.

As illustrated in **Figure** **8**a, each pre‐processed sMRI image (125 × 150 × 125) was diced into 150 non‐overlapped sMRI cubes (25 × 25 × 25) as the inputs of the *Ensemble 3DCNN* model. *Ensemble 3DCNN* model consisted of the base classifiers and a meta‐classifier (Figure 8b). Each base classifier was a 3DCNN consisting of seven layers, as illustrated in Figure 8c. The meta‐classifier contained one convolutional layer *(kernel size =1 × 12, stride = 1)* and a fully connected layer *(number of input channels = 64, number of output channels = 2)*. Each base classifier was independently trained using all small sMRI cubes from the same position of the training sMRI images with disease labels. 50% of sMRI cubes were randomly flipped along the *X* −, *Y* −, or *Z* − axis to increase data diversity during the training of the base classifiers. To avoid the possible negative effect caused by these poorly performed classifiers, base classifiers with recognition accuracy lower than 70% on the validation dataset were eliminated. Here, the 12 best‐performing base classifiers were retained among the total 150 base classifiers, and their output features of the penultimate layers (64‐dimension features) were concatenated as 768 (= 64 × 12) features to input into the meta‐classifier. The meta‐classifier was learned on the training dataset with the guide of the disease labels. As the final output of *Ensemble 3DCNN*, the output of the meta‐classifier was a 2D vector, in which these two elements corresponded to the probabilities regarding membership of the given input sMRI image to the class of AD or HC, that is, AD and HC predictive scores, respectively.

In fact, the proposed *Ensemble 3DCNN* was employed to better balance the two conflicting requirements of the model designed for analyzing the progression pattern of AD, that is, strong generalizability and good interpretability. It was expected that the progression patterns of AD embedded in the trained Ensemble 3DCNN and the datasets were intuitively disclosed while the model achieved satisfactory generalization capabilities in a data‐driven way.

The proposed *Ensemble 3DCNN* contained multiple base classifiers and a meta‐classifier. For achieving good generalizability, the base classifiers based on *3DCNN* were employed to extract the compelling features from the non‐overlapping small sMRI cubes all over the brain, while a meta‐classifier was built to allot the most appropriate weights to the base classifiers in a data‐driven manner. The advocated *Ensemble 3DCNN* integrated the prediction results of multiple base classifiers and learned a meta‐classifier to achieve satisfactory generalization capability during identifying the labels of unseen sMRI images finally. Thus, the output of the *Ensemble 3DCNN*, that is, the AD predictive score (probability) of an input image, was decided by both the features extracted by the base classifiers and the weights of the base classifiers allocated by the meta‐classifier. Compared with the other models, the proposed *Ensemble 3DCNN* achieved the best prediction performance, shown in Table S2, Supporting Information.

Each base classifier was trained with all the small sMRI cubes in the same position of the training images, as shown in Figure 8. Each base classifier learned to predict the AD probability of an unlabeled image while inputting the small sMRI cube in the same position as the one in which all the small sMRI cubes of the training images were utilized for training the base classifier from the unlabeled image. Since the same algorithm of *3DCNN* was used to train a specific base classifier based on the small sMRI cubes in a specific position of the same training images, the AD classification performance of a specific base classifier was directly and highly associated with the discriminative capability of the specific brain region (i.e., the specific position). Thus, the difference in the AD classification capability among the different base classifiers mainly resulted from the difference in the brain regions (positions) corresponding to these base classifiers. Hence, given an sMRI image of an AD patient as an input, there was no reason to doubt that the brain region corresponding to a base classifier with a higher AD predictive score as the output was much more likely to be neurodegenerative. Meanwhile, the brain region corresponding to a base classifier with a heavier weight allocated by the meta‐classifier was more important in determining the label of an sMRI image. In this way, the *Ensemble 3DCNN* was relatively simple in the model structure and had good interpretability.

On the one hand, the advocated *Ensemble 3DCNN* integrated the recognition ability of multiple base classifiers, exhibiting satisfactory performance in AD identification. On the other hand, the *Ensemble 3DCNN* facilitated intuitively differentiating among the brain regions with varied neurodegenerative risks and locating the high‐risk neurodegenerative brain regions. Both were the advantages of the proposed *Ensemble 3DCNN*.

### DL‐Extracted Neuroimaging Biomarker ***P*‐Score**

**Figure 9:**
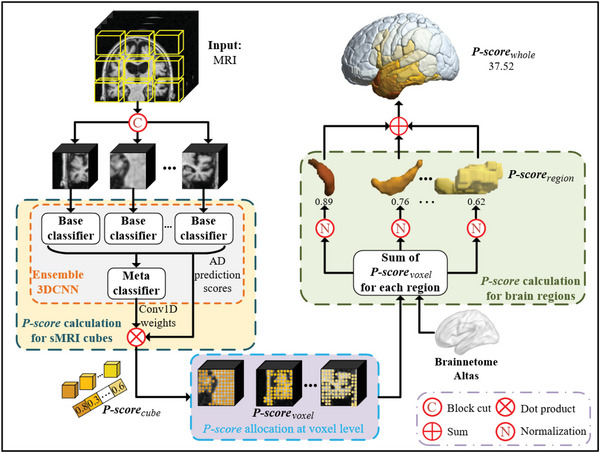
Illustration of *P*‐score calculation at different levels.

In this study, a neuroimaging biomarker, named *P*‐score, was derived to assess the degree of neurodegeneration in the brain of AD subjects. *P*‐score is a multi‐level measurement that can evaluate the neurodegeneration at the four levels (i.e., cube, voxel, region, and whole‐brain level). A higher *P*‐score value represents a higher degree of neurodegeneration. In **Figure** **9**, the relationship among *P*‐scores at the four levels is exhibited. Specifically, each sMRI image was divided into 150 non‐overlapping small cubes. *P*‐score of each small cube (*P*‐score_cube_) is defined as the weighted AD predictive score of its corresponding base classifier with the weights from the meta‐classifier. Here, the weights refer to the parameters of the first convolutional layer in the meta‐classifier. For sMRI cubes whose corresponding base classifiers were eliminated, their *P*‐score values were set as 0 because they were less relative to AD. For the rest sMRI cubes, *P*‐score_cube_ was evenly distributed to the voxels covered by the cerebral issue. The values of *P*‐score were set as 0 for the voxels without any cerebral tissue. Based on the Brainnetome Atlas,^[^ ^29^ ^]^ the human brain is parceled into 246 regions that reflect the whole‐brain's anatomical and functional connections. To calculate the value of *P*‐score of each brain region (*P*‐score_region_), the values of *P*‐score_voxel_ of all the voxels the region contains were first summed up and then, the sum was further divided by the size of the brain region (i.e., the number of the voxels it contains) to eliminate the effect of differences in the size of regions on *P*‐score_region_. For the sake of convenience, *P*‐score_region_ was finally scaled to the range of [0,1] using the Min‐Max normalization. Here, in the Min‐Max normalization, the minimum and maximum values were 1.157 × 10^−5^ and 0.1241, respectively. They are the maximal/minimal *P*‐score_region_ values (before normalization) of all brain regions in all the 720 sMRI images used for subsequent filtering with Criterion 3 and 2 and the pattern analysis of AD neurodegenerative progression, shown in Table 2 and Figure 2. At last, the *P*‐score_region_ of all brain regions were summed up to obtain the whole‐brain *P*‐score to evaluate the degree of neurodegeneration at the whole‐brain level, that is, *P*‐score_whole_(*i*) = ∑_*k*_ *P*‐score_region_(*i*, *k*). Here, *i* and *k* represented the index of an image and a brain region, respectively. *P*‐score_region_, the degree of neurodegeneration at the region level, was employed for analyzing AD progression from the standpoint of neuroimaging in this paper. By default, the *P*‐score means *P*‐score_region_ in the experimental section. The details on how to calculate *P*‐score at the different levels and the corresponding pseudo‐codes are presented in the Supporting Information. Plus, in the Supporting Information, the scatter diagram of *P*‐score_whole_ and AD *(softmax)* predictive score obtained by *Ensemble 3DCNN* for 638 AD sMRI images as shown in Table 2 and Figure 2 was plotted to exhibit the association between *P‐score* at whole‐brain level and AD probability output from *Ensemble 3DCNN*, that is, AD *(softmax)* predictive scores, to some degree.

### Connectivity Analysis

For each sMRI image, the neurodegenerative brain regions detected via *P*‐score were encoded in a binary vector, whose element values were equal to 1 if the corresponding brain regions were marked as neurodegenerative and 0 otherwise. The neighborhood information (adjacent regions) of brain regions was saved in a label set. DFS algorithm^[^ ^38^ ^]^ was employed to explore the connected components among the detected neurodegenerative brain regions for each AD sMRI image. The pseudo‐codes for connected component analysis are presented in the Supporting Information.

### Sequential Pattern Mining

As shown in Table 2, the 167 AD subjects who satisfied the above‐mentioned three criteria from the ADNI database^[^ ^27^ ^]^ were selected to form 167 longitudinal sMRI sequences for AD progression analysis. The neurodegenerative brain regions were labeled via the detection of *P*‐score. A standard sequential pattern mining algorithm, namely SPADE,^[^ ^39^ ^]^ was applied to explore the common neurodegenerative patterns in AD progression. The implementation steps such as sequence preparation, pattern mining and post‐screening, are detailed in the Supporting Information.

### Visualization of the Neurodegenerative Brain Regions

In this paper, a brain region with a higher *P*‐score value was considered as that with a higher degree of neurodegeneration.

In Figure 3, with the Brainnetome Atlas,^[^ ^29^ ^]^ brain regions were colored from light to dark according to their increasing *P*‐score value. In Figures 5, 6, 7, the brain regions with *P*‐score values higher than λ were selected as neurodegenerative regions. Here, λ = 0.73, which had been defined in the previous section. These neurodegenerative brain regions were then highlighted using different colors to display the neurodegenerative progression of AD in the brain in terms of sMRI.

### Statistical Analysis

1) *Pre‐processing of data*: sMRI images involved in this study were first pre‐processed using the Computational Anatomy Toolbox *(CAT12, dbm.neuro.uni‐jena.de/cat/)* with default setting for skull extraction, MNI space registration, and image smoothing. Each pre‐processed image was then normalized to a 121 × 145 × 121 image with values ranging from 0 to 1. The spatial resolution was 1.5 × 1.5 × 1.5 mm^3^ per voxel. Since 121 and 145 were not integral multiples of 25, each image with the size of 121 × 145 × 121 was reformatted to 125 × 150 × 125 using edge padding and zero filling for model training, performance test, and degeneration pattern analysis.
2) *Data information*: 2369 T1‐weighted structural MRI images of 1005 participants from *ADNI* and *OASIS* databases were involved in this study. The demographic and health‐related information of all the participants is summarized in Table 1. This work performed six analysis phases to explore the neurodegenerative patterns in AD, as shown in Figure 1. A data selection flowchart is illustrated in Figure 2 to clarify the datasets used in each phase. More detailed information of sMRI images involved in the six pattern analysis phases can be found in Table 2.
3) *Statistical methods for significance assessment*: Two‐sided testing was applied to assess the AD identification ability of the proposed *Ensemble 3DCNN* model and the radiomic features^[^ ^34^ ^]^ extracted by the Surfer Software Suite (https://www.freesurfer.net/). The resultant *P*‐value of each classification experiment is presented together with the AUC in the legend of each sub‐figure in Figure 4.
4) Software used for statistical analysis: *Ensemble 3DCNN* was implemented by TensorFlow 2.6.0 with NVIDIA GeForce RTX3090. DFS algorithm^[^ ^38^ ^]^ was employed for spatial/temporal‐spatial connectivity analysis of neurodegenerative brain regions among AD subjects. The common neurodegenerative patterns in AD progression were mined based on SPADE,^[^ ^39^ ^]^ which is a standard sequential pattern mining algorithm.

## Conflict of Interest

The authors declare no conflict of interest.

## Author Contributions

D.P., A.Z., and B.Y. designed the study. A.Z. coordinated the study. D.P., G.L., and B.Y. conducted the experiments. B.H., X.S., and T.J. reviewed the study design and data processing, and edited results interpretation and presentation. All authors drafted and revised the manuscript, and approved the final version of the submitted manuscript.

## Supporting information

Supporting Information

## Data Availability

Data from the Alzheimer's Disease Neuroimaging Initiative (ADNI) and the Open Access Series of Imaging Studies (OASIS) are available from the ADNI database (www.adni.loni.usc.edu) and the OASIS database (www.oasis‐brains.org) upon registration and compliance with the data usage agreement, respectively. Custom computer code used to produce results reported in the manuscript is available upon request.
