# Supplementary material for: Deep Learning for Brain MRI Confirms Patterned Pathological Progression in Alzheimer's Disease
Source: Adv Sci (Weinh). 2022 Dec 27;10(6):2204717. doi: 10.1002/advs.202204717 (PMC9951348; doi:10.1002/advs.202204717)
Supplement: Supplementary file 1 — Supporting Information [file ADVS-10-2204717-s001.pdf]

ADVANCED SCIENCE
Open Access

Supporting Information

for *Adv. Sci.*, DOI 10.1002/advs.202204717

Deep Learning for Brain MRI Confirms Patterned Pathological Progression in Alzheimer's Disease

Dan Pan, An Zeng, Baoyao Yang*, Gangyong Lai, Bing Hu, Xiaowei Song, Tianzi Jiang and Alzheimer's Disease Neuroimaging Initiative (ADNI)*

Supplementary Material – Deep Learning for Brain MRI Confirms Patterned Pathological Progression in Alzheimer's Disease

1 AD-related regions in the Brainnetome Atlas [1]

The abbreviations and locations of a part of the neurodegenerative brain regions associated with AD in the Brainnetome Atlas [1] are summarized in Table 1.

Brain Region			Gyrus (*Abbr.*, Name)	Lobe
Abbreviation (Left / Right)		Name		
L.mAmyg	*R.mAmyg*	Medial amygdala	*Amyg*, Amygdala	Subcortical nuclei
L.NAC	*R.NAC*	Nucleus accumbens	*BG*, Basal ganglia	Subcortical nuclei
L.lAmyg	*R.lAmyg*	Lateral amygdala	*Amyg*, Amygdala	Subcortical nuclei
L.vIa	*R.vIa*	Ventral agranular insula	*INS*, Insular gyrus	Insular lobe
L.vId/vIg	*R.vId/vIg*	Ventral dysgranular and granular insula	*INS*, Insular gyrus	Insular lobe
L.A38l	*R.A38l*	Lateral area 38	*STG*, Superior temporal gyrus	Temporal lobe
L.TI	*R.TI*	Area TI (temporal agranular insular cortex)	*PhG*, Parahippocampal gyrus	Temporal lobe
L.rHipp	*R.rHipp*	Rostral hippocampus	*Hipp*, Hippocampus	Subcortical nuclei
L.A28/34	*R.A28/34*	Area 28/34 (EC, entorhinal cortex)	*PhG*, Parahippocampal gyrus	Temporal lobe
L.vmPu	*R.vmPu*	Ventromedial putamen	*BG*, Basal ganglia	Subcortical nuclei
L.A35/36r	*R.A35/36r*	Rostral area 35/36	*PhG*, Parahippocampal gyrus	Temporal lobe
L.A20r	*R.A20r*	Rostral area 20	*ITG*, Inferior temporal gyrus	Temporal lobe

Table 1: Detailed information on a part of neurodegenerative brain regions associated with AD in the Brainnetome Atlas [1]

2 AD Classification Performance of Ensemble 3D Convolutional Neural Networks (*Ensemble 3DCNN*)

As a proof of concept, we posit the machine learning model could effectively distinguish sMRI images of AD subjects from those of the healthy controls (HC). The neuroimaging biomarker (P-**score**) is detected in the sMRI images on the basis of a proposed machine learning model, named *Ensemble 3DCNN*. In this section, before we demonstrate the effectiveness of P-**score** in indicating the degree of neurodegenerative in the AD brain, the capability of the *Ensemble 3DCNN* to identify AD subjects from the healthy controls (HC) is verified. With the three evaluation metrics, i.e., classification accuracy (ACC), Area under the curve (AUC), and Matthews Correlation Coefficient (MCC) [2], *Ensemble 3DCNN* is evaluated on the validation and testing datasets retrieved from the ADNI database[3] and OASIS database [4], respectively. The experimental results are detailed in Table 2. The performance of *Ensemble 3DCNN* is superior to other machine learning models *(PCA+SVM [5],3D-SENet [6], and wH-FCN [7])*. More importantly, with *Ensemble 3DCNN*, an AUC value of **88.4%** is achieved on the testing dataset retrieved from the OASIS database [4], which verifies the generalization ability of *Ensemble 3DCNN* when it is applied across databases.

	Validation dataset retrieved from the ADNI database [3]			Testing dataset retrieved from the OASIS database [4]		
Method	ACC	AUC	MCC	ACC	AUC	MCC
PCA+SVM [5]	0.772	– –	– –	0.726	– –	– –
3D-SENet [6]	0.851	0.920	0.681	0.786	0.851	0.514
wH-FCN [7]	0.818	0.880	0.641	0.790	0.835	0.533
Ensemble 3DCNN	0.865	0.927	0.743	0.820	0.884	0.600

Table 2: Comparison results of four models on two datasets in the task of classifying between AD and HC.

3 Probability map of brain neurodegeneration in the AD population

In our paper, some common neurodegenerative brain regions in AD are extracted, as listed in Table 3 in the main text of this manuscript. According to the frequency (neurodegenerative probability) of each brain region, we draw a probability map in Figure 1 to show the distribution of the common neurodegenerative regions in the brain intuitively. It can be observed that the common degenerative regions locate in the area around the amygdala, nucleus accumbens, agranular insular cortex, and hippocampus, which are approximately corresponding to the isocortex, basal magnocellular complex, and transentorhinal regions in the brain.

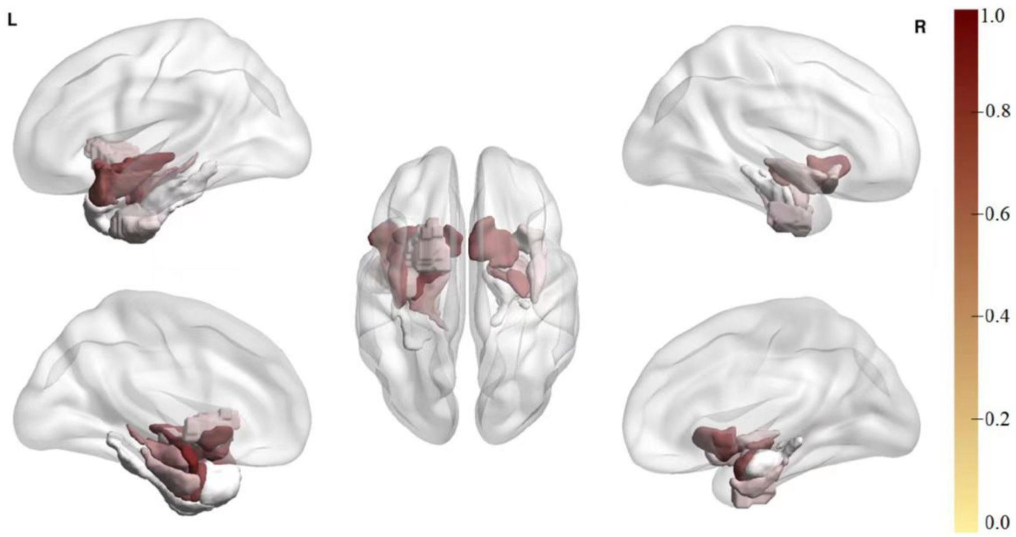

Figure 1: Probability map of the brain neurodegeneration in the AD population.

4 Scatter diagrams of the P-*score*$_{whole}$ and *MMSE* [8] values

It is found that the P-*score*$_{whole}$ is more sensitively indicates less severe cognitive impairment *(MMSE [9] > 20)* in comparison with the existing radiomic features[10]. The correlation between P-*score*$_{whole}$ and *MMSE [9]* value is shown in the scatter diagram of Figure 2. It is noticeable that with the decrease in *MMSE* value, the smallest P-*score*$_{whole}$ value among all AD sMRI images corresponding to the same *MMSE* value increases.
Among the subjects with P-*score*$_{whole}$ lower than 40, only a small percentage (less than **16**%) of subjects are in moderate or worse cognitive impairment (i.e., $(MMSE < 20)$). The *Chi-Squared* test is adopted to verify this observation among AD sMRI images. Hypothesize that $MMSE \geq 20$ is independent of P-*score*$_{whole} < 40$. The *Chi-Squared* value is 7.15, which means the probability that $MMSE \geq 20$ and P-*score*$_{whole} < 40$ are irrelevant is lower than 2.5% *(Freedom Degree=1)*. When P-*score*$_{whole}$ is rounded to the nearest integer, the Pearson's correlation coefficient between a P-*score*$_{whole}$ and the corresponding smallest *MMSE* value is -0.80 *(p-value*$=3.06 \times 10^{-6}$*)*, indicating a fairly strong negative relationship between P-*score*$_{whole}$ and the lower bound of *MMSE*. Therefore, P-*score*$_{whole}$ can be used as a preliminary measurement to exclude moderate or worse cognitive impairment. For comparison, we also measure the eight radiomic features [10] for sMRI images and plot the scatter diagram of these features and *MMSE* values. The eight diagrams corresponding to the eight measures are displayed in Figure 3. Unlike P-*score*$_{whole}$, the radiomic features *(such as gray matter volume, cortical area, and cortical thickness)* take similar values for the AD sMRI images in different cognitive impairment levels. The reason for this result might be the individual diversity in brain size among AD subjects.

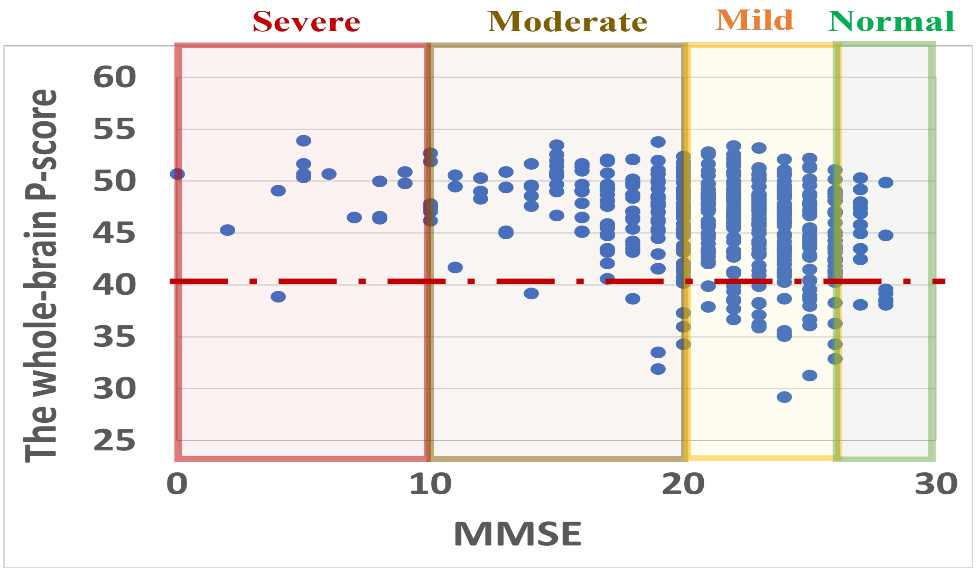

Figure 2: Scatter diagram of $P\text{-}score_{whole}$ and $MMSE$ [9].

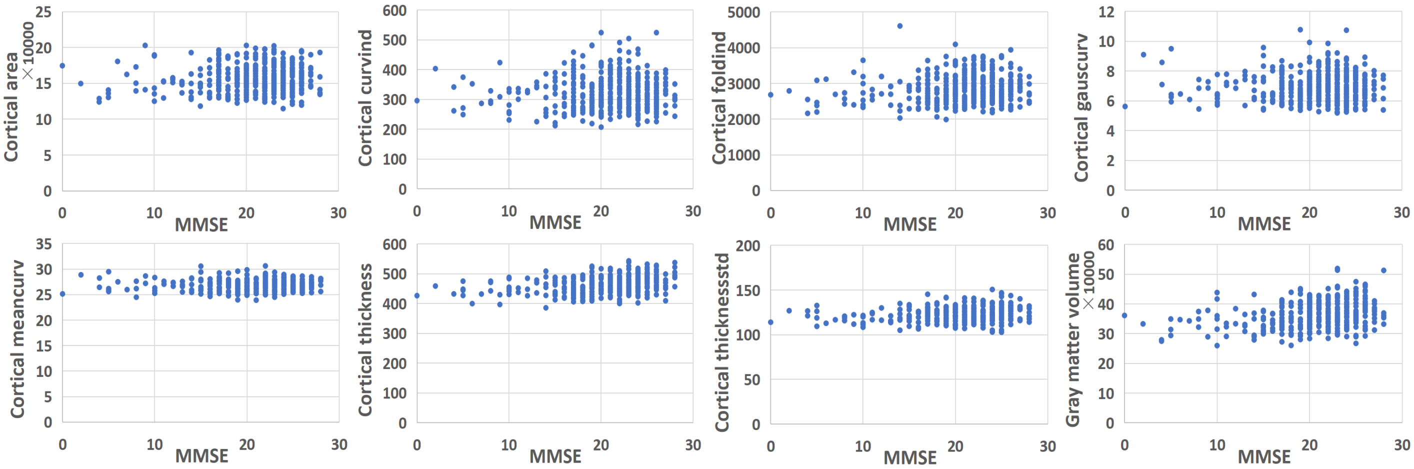

Figure 3: Scatter diagrams of $MMSE$ values [8] and each of eight radiomic features .

5 P-score calculation at the four levels

In this paper, we derive a neuroimaging biomarker, named P**-score**, to assess the degree of neurodegeneration for AD subjects. P**-score** is a multi-level measurement that can evaluate the neurodegeneration at the four levels (cube, voxel, region, and whole-brain level). The higher $P\text{-}score$ value represents the higher degree of neurodegeneration.

1) P-score calculation for sMRI cubes:

Each sMRI image is divided into non-overlapping small cubes. $P\text{-}score$ of each small cube is defined as the weighted AD predictive score of its corresponding base classifier with the weights from the meta-classifier. Mathematically, $P\text{-}score_{cube}(i,c) = w_c \cdot p_{i,c}$, where i and c are indexes of sMRI images and cubes, respectively. $p_{i,c}$ denotes the AD predictive result *(softmax score)* obtained by the c-th base classifier of *Ensemble 3DCNN* with the c-th cube in the i-th sMRI image as an input. w_c represents the contribution of the c-th base classifier to AD predictive scores, i.e., the final decision of *Ensemble 3DCNN*, and is a parameter in the parameter vector in the meta-classifier of *Ensemble 3DCNN*. Here, the weights of base classifiers whose accuracies in the validation dataset are less than 70% are set to 0 to ensure the reliability of neurodegeneration measurement.

2) P-score allocation at voxel level:

In each sMRI cube, we evenly distribute $\boldsymbol{P\text{-}score}_{cube}$ to the voxels covered by the cerebral tissue. We denote $P\text{-}score$ of the j-th voxel, i.e., $voxel_j$, in the i-th sMRI image as $P\text{-}score_{voxel}(i,j)$. That is, P-

$score_{voxel}(i,j) = P\text{-}score_{cube}(i,c)/|\boldsymbol{S}_{i,c}^{act}|$. Here, $|\boldsymbol{S}_{i,c}^{act}|$ denotes the number of voxels covered by the cerebral tissue within an sMRI cube $\boldsymbol{X}_c^i$, i.e., the c-th cube in the i-th sMRI image, where $voxel_j$ is located, i.e., $voxel_j \in \boldsymbol{X}_c^i$.

3) P-score calculation for brain regions:

Based on the Brainnetome Atlas [1], the human brain is parceled into 246 regions that reflect the whole brain's anatomical and functional connections. P-*score* of each brain region (before normalization) is the sum of $P\text{-}score_{voxel}(i,j)$ of each voxel it contains. We further divided $\boldsymbol{P\text{-}score}_{region}$ by the size of the brain region (i.e., the number of voxels in the brain region) to eliminate the effect of differences in the size of regions. For observation convenience, $\boldsymbol{P\text{-}score}_{region}$ (before normalization) is scaled to the range of $[0,1]$ using the Min-Max normalization[a]. The calculation of $\boldsymbol{P\text{-}score}_{region}$ (after normalization) for each brain region can be written as

$$\boldsymbol{P\text{-}score}_{region}(i,k) = Norm(\frac{1}{|\boldsymbol{\Omega}_k|}\sum_{voxel_j \in \boldsymbol{\Omega}_k} \boldsymbol{P\text{-}score}_{voxel}(i,j)) \tag{1}$$

where i, j and k represent the index of an image, a voxel and a region, respectively; $\boldsymbol{\Omega}_k$ denotes a set of voxels within the k-th brain region in the Brainnetome Atlas, and $|\boldsymbol{\Omega}_k|$ stands for the number of voxels within the k-th brain region in the Brainnetome Atlas. $Norm(\cdot)$ represents a Min-Max normalization function. By default, both $\boldsymbol{P\text{-}score}$ and $\boldsymbol{P\text{-}score}_{region}$ represent $\boldsymbol{P\text{-}score}_{region}$ (after normalization) .

4) The whole-brain P-*score*:

Finally, we sum up $\boldsymbol{P\text{-}score}_{region}$ of all regions to get the whole-brain P-*score* to evaluate the degree of neurodegeneration at the whole-brain level.

Thus, according to those mentioned above,

$$\begin{aligned}
\boldsymbol{P} - \boldsymbol{score}_{whole}(i) &= \sum_k \boldsymbol{P} - \boldsymbol{score}_{region}(i,k) \\
&= \sum_k [Norm(\frac{1}{|\boldsymbol{\Omega}_k|}\sum_{voxel_j \in \boldsymbol{\Omega}_k} \boldsymbol{P} - \boldsymbol{score}_{voxel}(i,j))] \\
&= \sum_k [Norm(\frac{1}{|\boldsymbol{\Omega}_k|}\sum_{voxel_j \in \boldsymbol{\Omega}_k, voxel_j \in \boldsymbol{X}_c^i} \boldsymbol{P} - \boldsymbol{score}_{cube}(i,c)/|\boldsymbol{S}_{i,c}^{act}|] \\
&= \sum_k [Norm(\frac{1}{|\boldsymbol{\Omega}_k|}\sum_{voxel_j \in \boldsymbol{\Omega}_k, voxel_j \in \boldsymbol{X}_c^i} (w_c \cdot p_{i,c})/|\boldsymbol{S}_{i,c}^{act}|]
\end{aligned} \tag{2}$$

The $\boldsymbol{P\text{-}score}_{region}$ at the region level is used for subsequent pattern analysis in this paper.

Pseudo-codes for P-**score** calculation are presented in Algorithm 1.

[a] In the Min-Max normalization, the minimum and maximum values are 1.157×10^{-5} and 0.1241, respectively. They are the maximal/minimal $\boldsymbol{P\text{-}score}_{region}$ value (before normalization) of all brain regions in all the 720 sMRI images used for subsequent filtering with Criterion 3 and 2 and pattern analysis of AD neurodegenerative progression, shown in Table 2 in the main text of this article.

Algorithm 1 P**-score** calculation for each sMRI image

Input: The i-th sMRI image, which is divided into C small cubes, $\boldsymbol{X}^i = \{\boldsymbol{X}_c^i\}_{c=1,\ldots,C}$; The base classifiers of the *Ensemble 3DCNN*, $\boldsymbol{\mathcal{M}} = \{\mathcal{M}_c\}_{c=1,\ldots,C}$; The weights for each cube (base classifier), $\boldsymbol{w} = \{w_c\}_{c=1,\ldots,C}$; The Brainnetome Atlas [1], $\boldsymbol{\Omega} = \{\boldsymbol{\Omega}_k\}_{k=1,\ldots,K}$, $K = 246$.

Output: For the i-th sMRI image, P-*score* of each brain region, $\boldsymbol{P\text{-}score}_{region}(i) \in \mathcal{R}^{1\times K}$, and the whole-brain P-*score*, $P\text{-}score_{whole}(i)$.

function GET_P-SCORE($\boldsymbol{X}^i$, $\mathcal{M}$, $\boldsymbol{w}$, $\boldsymbol{\Omega}$)
  Initialize $\boldsymbol{P\text{-}score}_{region}(i)$ as a K-dimension zero vector.
  for Each $\mathcal{M}_c$ in $\boldsymbol{\mathcal{M}}$ **do**
    $p_{i,c} = \mathcal{M}_c(\boldsymbol{X}_c^i)$; *{Predict the probability of AD for each small cube $\boldsymbol{X}_c^i$ in the i-th sMRI image using the base classifier $\mathcal{M}_c$}*
    $P\text{-}score_{cube}(i,c) = w_c \cdot p_{ic}$; *{Calculate P-score for each small cube $\boldsymbol{X}_c^i$ in the i-th sMRI image}*
    for Voxel j within $\boldsymbol{X}_c^i$ **do**
      if Voxel j is covered by the cerebral tissue **then**
        $P\text{-}score_{voxel}(i,j) = P\text{-}score_{cube}(i,c)/|\boldsymbol{S}_{i,c}^{act}|$; *{Allocate $P\text{-}score_{cube}(i,c)$ at the voxel level, $|\boldsymbol{S}_{i,c}^{act}|$ denotes the number of voxels covered by the cerebral tissue within $\boldsymbol{X}_c^i$, the c-th cube in the i-th sMRI image}*
      end if
    end for
  end for
  for Each $\boldsymbol{\Omega}_k$ in $\boldsymbol{\Omega}$ **do**
    for Voxel j within $\boldsymbol{\Omega}_k$ **do**
      $P\text{-}score_{region}(i,k) \mathrel{+}= P\text{-}score_{voxel}(i,j)$;
    end for
    $P\text{-}score_{region}(i,k) \mathrel{/}= |\boldsymbol{\Omega}_k|$; *{Calculate P-score at the region level (before normalization)}*
    $P\text{-}score_{region}(i,k) = Norm(P\text{-}score_{region}(i,k))$; *{Calculate P-score at the region level (after normalization)}*
  end for
  $P\text{-}score_{whole}(i) = \sum_k P\text{-}score_{region}(i,k)$;
  return $\boldsymbol{P\text{-}score}_{region}(i)$, $P\text{-}score_{whole}(i)$
end function

6 Associations between P-*score* at whole-brain level and AD predictive scores obtained by *Ensemble 3DCNN*

Since P**-score** is a neuroimaging biomarker derived from the results of the trained *Ensemble 3DCNN*, for each sMRI image, its P-*score* at whole-brain level should be associated with its AD probability output from *Ensemble 3DCNN*, i.e., AD *(softmax)* predictive scores. In Figure 4, we plot the scatter diagram of $P\text{-}score_{whole}$ and AD *(softmax)* predictive score obtained by *Ensemble 3DCNN* for 638 AD sMRI images in the analysis dataset, as shown in Table 2 in the main text of this article. Their correlation coefficient *(Pearson coefficient)* is 0.60 with *p-value* of 3.843×10^{-63} *(<0.01)*, indicating a moderately to strongly positive correlation. In Figure 4(b), the lower bound of $P\text{-}score_{whole}$ increases with the increase of AD predictive score. Moreover, many scattered points with $P\text{-}score_{whole}$ larger than 40 are gathered around the vertical line with the AD predictive score of 1. This is because *Ensemble 3DCNN* is trained by the beginning sMRIs among each longitudinal AD sMRI sequence and the outputs of *Ensemble 3DCNN* are compressed by the softmax function, resulting in the fact that the AD predictive scores of many subsequent AD sMRI images in a longitudinal AD sMRI sequence are almost equal to 1 via the compressive saturation response of the softmax function. We stretch the axis of the AD predictive score to observe its correlation with $P\text{-}score_{whole}$ in its range of [0.99, 1]. As illustrated in Figure 4(b), the moderately to strongly positive correlation between $P\text{-}score_{whole}$ and AD predictive score in its range of [0.99, 1] still holds and remains statistically significant. In contrast, $P\text{-}score_{whole}$ is based on the original output results from base classifiers of *Ensemble 3DCNN*, which provide relatively distinguishable and contrastive values for the AD sMRI images in a longitudinal AD sMRI sequence.

7 Connected component analysis of neurodegenerative brain regions

For each sMRI image, we save the neurodegenerative status of the brain regions detected with P**-score** as a binary vector $\boldsymbol{v}_i \in \mathcal{R}^{1\times K}$, where $\boldsymbol{v}_i(k) = 1$ if the k-th brain region is labeled as neurodegeneration in the i-th sMR image. The neighborhood around each brain region is saved as a label set, denoted as

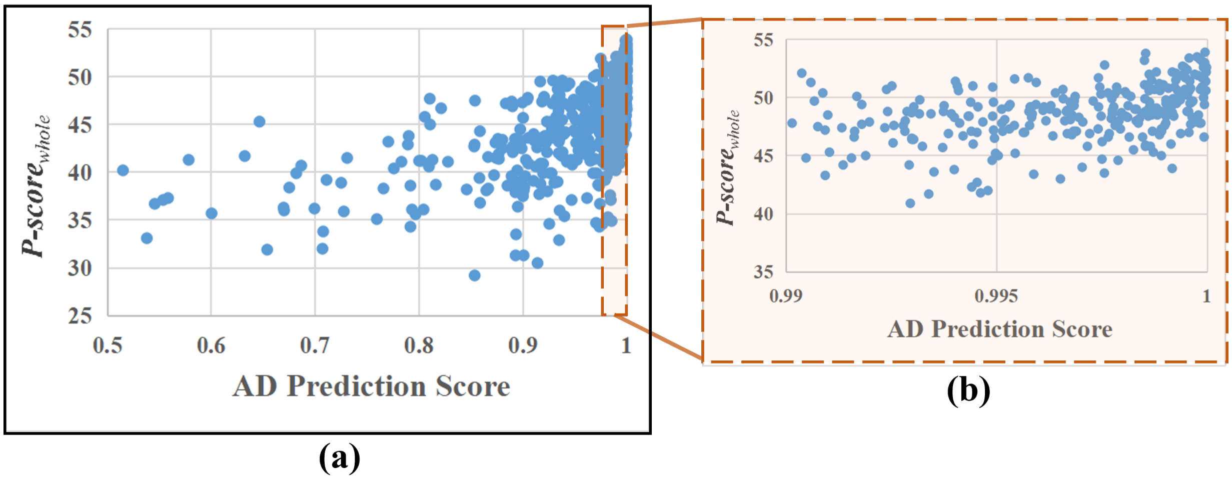

Figure 4: Scatter diagram of $P\text{-}score_{whole}$ and AD predictive score *(predictions via softmax function)* with its range in [0.5, 1] (a) and [0.99, 1] (b).

$\mathcal{B} = \{\boldsymbol{NeighborList}(k)\}_{k=1,\ldots K}$, where $\boldsymbol{NeighborList}(k)$ saves the adjacent brain regions of the k-th region.

Depth First Search (DFS) algorithm [11] is employed to explore the connected components in the detected neurodegenerative brain regions, i.e., $\boldsymbol{v}_i$, for each AD sMRI image. The pseudo-codes for connected component analysis are presented in Algorithm 2. When analyzing the spatial-temporal connectivity for each AD subject, each AD subject's longitudinal 3D sMRI images are concatenated along the time axis to generate a 4D sample for connected component analysis. The binary vector $\boldsymbol{v}_i$ and its corresponding neighbour $NeighborList(k)$ now record the neurodegenerative brain regions in the 4D sample and the spatial-temporal adjacent regions of each neurodegenerative brain region, respectively. The algorithm for the spatial-temporal connected component analysis is similar to Algorithm 2.

Algorithm 2 Connected component analysis of the neurodegenerative brain regions for each sMRI image

Input: A binary vector consisting of 1s (yes) and 0s (no), i.e., $\boldsymbol{v}_i$, which saves the status on whether or not each brain region is neurodegenerative in the i-th test sMRI image; The neighborhood information in the Brainnetome Atlas [1], $\mathcal{B} = \{\boldsymbol{NeighborList}(k)\}_{k=1,\ldots K}$.

Output: Number of the connected components, $Count(i)$; Members in the connected components, $\boldsymbol{ComponentList}(i)$

function COUNT_CONNECTEDCOMPONENT($\boldsymbol{v}_i$, $\mathcal{B}$)
    Initialization: $Count(i) = 0, \boldsymbol{visit} = \varnothing$;   *{Create a set **visit** to save the brain regions which have been visited}*
    function GET_REGION_CONNECT($Region$)
        Initialization: $\boldsymbol{CONNECT\_LIST} = \{Region\}$ *{Member in the connected brain regions}*
        $\boldsymbol{visit} \longleftarrow Region$ *{Insert Region into **visit**, mean that Region has been visited}*
        for Each brain region $j \in \boldsymbol{NeighborList}(Region)$ **do**
            if $\boldsymbol{v}_i(j) == 1$ && j not in $\boldsymbol{visit}$ **then**
                $\boldsymbol{NewRegion}$ = GET_REGION_CONNECT(j)   *{Search for the next unsearched brain regions}*
                $\boldsymbol{CONNECT\_LIST}$ extend $\boldsymbol{NewRegion}$
            end if
        end for
        return $\boldsymbol{CONNECT\_LIST}$
    end function
    for Each brain region $k \in [1, ..., K]$ **do**
        if $\boldsymbol{v}_i(k) == 1$ && $\boldsymbol{v}_i(k)$ not in $\boldsymbol{visit}$ **then**
            $\boldsymbol{ComponentList}(i)[Count(i)]$ = GET_REGION_CONNECT(k)
            $Count(i) += 1$
        end if
    end for
    return $Count(i), \boldsymbol{ComponentList}(i)$
end function

8 Mining sequential patterns on neurodegeneration in AD progression

For each sMRI images from the 167 AD subjects in the analysis dataset retrieved from ADNI database [3] as shown in Table 2 in the main text of this article, we label the neurodegenerative brain regions with the detection of P-**score**. Here, the 167 AD subjects take MRI examinations at multiple time points, forming 167 longitudinal sMRI sequences. Each sequence is denoted as $\boldsymbol{S}_i = \{\boldsymbol{R}_i^1 \longrightarrow \cdots \longrightarrow \boldsymbol{R}_i^{t_i} \cdots \longrightarrow \boldsymbol{R}_i^{T_i}\}$, where $\boldsymbol{R}_i^{t_i} = \{r_1^{t_i}, r_2^{t_i}, \cdots, r_{k_{t_i}}^{t_i}, \cdots, r_{K_{t_i}}^{t_i}\}$ is an item-set recording the neurodegenerative brain regions in the t_i-th sMRI image of the longitudinal sequence $\boldsymbol{S}_i$. $r_{k_{t_i}}^{t_i}$ represents the k_{t_i}-th neurodegenerative brain region in $\boldsymbol{R}_i^{t_i}$. K_{t_i} denotes the number of neurodegenerative brain regions in the t_i-th sMRI image in the sequence $\boldsymbol{S}_i$, and T_i is the number of sMRI images in the sequence $\boldsymbol{S}_i$. We search for the sequential patterns that frequently occur in these sequences to explore the patterns of neurodegeneration in AD progression. Three main steps are included: 1) preparing longitudinal sequences, 2) mining frequent sequential patterns, and 3) post-screening frequent sequential patterns.

1) Preparing longitudinal sequences Medical studies[12] have indicated that AD tends to progress in a continuous and irreversible manner. For each longitudinal sequence recording neurodegenerative brain regions, we add the regions in the antecedent item-sets to the consequent item-sets to preserve the irreversibility, if necessary. For each sequence $\boldsymbol{S}_i$, we have $\boldsymbol{R}_i^p \subseteq \boldsymbol{R}_i^q$, if $q > p$. For the sake of simplicity in expression, regions that have appeared in the antecedent item-set are omitted in the consequent item-set for the same sequence. The sequence $\boldsymbol{S}_i$ is updated as $\boldsymbol{S}_i = \{\boldsymbol{R}_i^1 \longrightarrow \{\boldsymbol{R}_i^{t_2}/\boldsymbol{R}_i^{t_1}\} \longrightarrow \{\boldsymbol{R}_i^{t_3}/\{\boldsymbol{R}_i^{t_2} \cup \boldsymbol{R}_i^{t_1}\}\} \longrightarrow \cdots \longrightarrow \{\boldsymbol{R}_i^{T_i}/\{\boldsymbol{R}_i^{T_i-1} \cup \cdots \cup \boldsymbol{R}_i^{t_2} \cup \boldsymbol{R}_i^{t_1}\}\}\}$. Finally, the original long sequences are separated into subsequences $\{\tilde{\boldsymbol{S}}_j\}_{j=1,2,\ldots,n}$, where n represents the total number of subsequences. Each subsequence $\tilde{\boldsymbol{S}}_j = \{\boldsymbol{R}_j^p \longrightarrow \boldsymbol{R}_j^q\}$ contains only one antecedent item-set $\boldsymbol{R}_j^p$ and one consequent item-set $\boldsymbol{R}_j^q$. We save the subjects' IDs corresponding to these subsequences and use them as the inputs for mining frequent sequential patterns in the AD progression.

2) Mining frequent sequential patterns The *SPADE algorithm*[13] is adopted to mine the frequent sequential patterns among the neurodegenerative brain region sequences, i.e., $\{\tilde{\boldsymbol{S}}_j\}_{j=1,2,\ldots,n}$, where n represents the number of subsequences. The support is set to 0.05 to obtain as many frequent items as possible in an acceptable time period. More than 163,000 frequent sequential patterns are extracted.

3) Post-screening frequent sequential patterns A series of post-screening processes are applied to explore the effective neurodegeneration patterns from thousands of frequent sequential patterns.
Here, the support for each frequent sequential pattern is updated with the number of subjects that this sequential pattern is applicable to. The sequential patterns with small support (< 20) are considered infrequent items, which are excluded. Finally, for two sequential patterns ($\{\boldsymbol{R}^{p1} \longrightarrow \boldsymbol{R}^{q1}\}$ and $\{\boldsymbol{R}^{p2} \longrightarrow \boldsymbol{R}^{q2}\}$) that share the same supporting subject IDs and are the same in terms of consequent item sets ($\boldsymbol{R}^{q1} = \boldsymbol{R}^{q2}$), we further analyze their antecedent item sets $\boldsymbol{R}^{p1}$ and $\boldsymbol{R}^{p2}$. When $\boldsymbol{R}^{p1} \subseteq \boldsymbol{R}^{p2}$, only the sequential patterns of $\{\boldsymbol{R}^{p2} \longrightarrow \boldsymbol{R}^{q2}\}$ are retained in the final result. After the post-screening, ***87*** frequent sequential patterns *(with a supporting rate greater than **16%**)* that reflect the AD progression patterns are extracted from the longitudinal sequences of neurodegenerative brain regions. The most common sequential patterns are listed in Table 3. Table 3 also reports the frequency *(supporting rate[b])* of each sequential pattern in the OASIS database [4] to verify the generalization of the neurodegenerative patterns in the ADNI database detected via P-**score**. In the OASIS database [4], 26 AD subjects have longitudinal sMRI image sequences and are correctly identified by *ensemble 3DCNN*. Among these 26 AD subjects, 22 subjects *(with 46 sMRI images)*, as shown in Table 2 in the main text of this article, contain at least one neurodegenerative brain region detected by P-**score** and are selected for mining frequent sequential patterns. Although the supporting rates of frequent sequential patterns in the OASIS database

[b]The calculation of λ_{oasis} for degenerative region detection in the OASIS database is the same as that in the ADNI database[3]. In specific, $\lambda_{oasis} = mean + 2std = 0.733$, which is determined by the mean and standard deviation (std) of all brain regions' P-**score** among the AD sMRI images correctly identified by the *Ensemble 3DCNN* in the OASIS database[4].

[4] are lower than those in the ADNI database[3], most *(**92%**, 80 of 87 frequent sequential patterns)* frequent sequential patterns mined in the ADNI database [3] can be detected in the OASIS database[4] as well. The average supporting rate of the top ***20*** frequent sequential patterns can reach ***13.33%*** in the OASIS database[4].

No.	Frequent Sequential Patterns	Frequency in ADNI [3] database	Frequency OASIS [4] database
1	*{L.NAC} ⟶ { #, L.A28/34}*	28.1%	11.1%
2	*{L.mAmyg, L.NAC} ⟶ { #, L.A28/34}*	27.0%	11.1%
3	*{L.NAC} ⟶ { #, R.A28/34}*	24.0%	16.7%
4	*{L.vIa, L.lAmyg} ⟶ { #, L.vmPu}*	23.4%	11.1%
5	*{L.mAmyg} ⟶ { #, L.vmPu}*	23.4%	11.1%
6	*{L.mAmyg} ⟶ { #, L.rHipp}*	23.4%	16.7%
7	*{L.vIa, L.mAmyg, L.lAmyg, L.NAC} ⟶ { #, L.A28/34}*	23.4%	5.6%
8	*{L.mAmyg, L.NAC} ⟶ { #, L.vmPu}*	22.8%	11.1%
9	*{R.NAC} ⟶ { #, R.A28/34}*	22.8%	22.2%
10	*{L.NAC} ⟶ { #, L.rHipp}*	22.8%	11.1%
11	*{L.NAC} ⟶ { #, R.mAmyg}*	22.8%	5.6%
12	*{R.mAmyg} ⟶ { #, R.A28/34}*	22.8%	27.8%
13	*{L.mAmyg, L.NAC} ⟶ { #, R.A28/34}*	22.8%	16.7%
14	*{L.vIa, L.mAmyg, L.lAmyg } ⟶ { #,L.vmPu}*	22.8%	11.1%
15	*{L.A38l, L.TI, L.vIa, L.vId/vIg, L.mAmyg, L.lAmyg} ⟶ { #, L.vmPu}*	22.2%	11.1%
16	*{L.NAC} ⟶ { #, R.NAC}*	22.2%	5.6%
17	*{L.NAC, R.NAC} ⟶ { #, R.A28/34}*	22.2%	16.7%
18	*{L.vIa, L.mAmyg, L.lAmyg, L.NAC} ⟶ { #, L.vmPu}*	22.2%	11.1%
19	*{R.mAmyg, R.NAC} ⟶ { #, R.A28/34}*	22.2%	22.2%
20	*{L.A38l, L.A35/36r, L.TI, L.vIa, L.vId/vIg, L.mAmyg, L.lAmyg} ⟶ { #, L.vmPu}*	21.6%	11.1%
21	*{L.mAmyg, R.mAmyg, L.NAC} ⟶ { #, L.A28/34}*	21.6%	5.6%
22	*{R.mAmyg, L.NAC, R.NAC} ⟶ { #, R.A28/34}*	21.6%	16.7%
23	*{L.mAmyg, L.NAC, R.NAC} ⟶ { #, R.A28/34}*	21.6%	16.7%
24	*{L.mAmyg, L.NAC} ⟶ { #, L.rHipp}*	21.6%	11.1%
25	*{L.TI, L.vIa, L.vId/vIg, L.mAmyg, L.lAmyg, L.NAC} ⟶ { #, L.A28/34}*	21.6%	5.6%
26	*{L.A38l, L.TI, L.vIa, L.vId/vIg, L.mAmyg, L.lAmyg, L.NAC} ⟶ { #, L.vmPu}*	21.6%	11.1%
27	*{L.mAmyg} ⟶ { #, R.mAmyg}*	21.0%	5.6%
28	*{L.mAmyg, R.mAmyg, L.NAC, R.NAC} ⟶ { #, R.A28/34}*	21.0%	16.7%
29	*{L.A38l, L.A35/36r, L.TI, L.vIa, L.vId/vIg, L.mAmyg, L.lAmyg, L.NAC} ⟶ { #, L.vmPu}*	21.0%	11.1%
30	*{L.mAmyg} ⟶ { #, R.NAC}*	20.4%	5.6%
31	*{L.vIa, L.lAmyg} ⟶ { #, L.rHipp}*	20.4%	16.7%
32	*{L.mAmyg, L.NAC} ⟶ { #, R.mAmyg}*	20.4%	5.6%
33	*{L.A35/36r, L.TI, L.vIa, L.vId/vIg, L.mAmyg, L.lAmyg, L.NAC} ⟶ { #, L.A28/34}*	20.4%	5.6%
34	*{L.A38l, L.TI, L.vIa, L.vId/vIg, L.mAmyg, L.lAmyg, L.NAC} ⟶ { #, L.A28/34}*	20.4%	5.6%
35	*{L.mAmyg, L.NAC, R.NAC} ⟶ { #, L.A28/34}*	19.8%	5.6%
36	*{L.vIa, L.mAmyg, R.mAmyg, L.lAmyg, L.NAC} ⟶ { #, L.A28/34}*	19.8%	0%
37	*{L.mAmyg, L.NAC} ⟶ { #, R.NAC}*	19.8%	5.6%
38	*{L.A38l, L.A35/36r, L.TI, L.vIa, L.vId/vIg, L.mAmyg, L.lAmyg, L.NAC} ⟶ { #, L.A28/34}*	19.8%	5.6%
39	*{L.A38l, L.A35/36r, L.TI, L.vIa, L.vId/vIg, L.mAmyg, L.lAmyg, L.rHipp, L.NAC} ⟶ { #, L.vmPu}*	19.2%	5.6%
40	*{L.mAmyg, R.mAmyg, L.NAC} ⟶ { #, L.vmPu}*	19.2%	5.6%
41	*{L.TI, L.vIa, L.lAmyg} ⟶ { #, L.rHipp}*	19.2%	16.7%
42	*{L.vIa, L.mAmyg, L.lAmyg} ⟶ { #, L.rHipp}*	19.2%	11.1%
43	*{L.vIa, L.mAmyg, R.mAmyg, L.lAmyg, L.NAC} ⟶ { #, L.vmPu}*	18.6%	5.6%
44	*{L.TI, L.vIa, L.mAmyg, L.lAmyg} ⟶ { #, L.rHipp}*	18.6%	11.1%
45	*{L.TI, L.vIa, L.vId/vIg, L.lAmyg} ⟶ { #, L.rHipp}*	18.0%	16.7%
46	*{L.mAmyg} ⟶ { #, R.lAmyg}*	18.0%	11.1%
47	*{L.vIa, L.lAmyg} ⟶ { #, R.A28/34}*	18.0%	11.1%
48	*{L.mAmyg, R.mAmyg, L.NAC, R.NAC} ⟶ { #, L.A28/34}*	18.0%	5.6%
49	*{L.TI, L.vIa, L.vId/vIg, L.mAmyg, R.mAmyg, L.lAmyg, L.NAC} ⟶ { #, L.A28/34}*	18.0%	0%
50	*{L.A38l, L.TI, L.vIa, L.vId/vIg, L.mAmyg, R.mAmyg, L.lAmyg, L.NAC} ⟶ { #, L.vmPu}*	18.0%	5.6%

Continued

Table 3: Frequent sequential patterns of the neurodegenerative brain regions in the longitudinal sMRI image sequences of AD subjects.
Symbol # represents the elements in the antecedent.

Table 3 Continued

No.	Frequent Sequential Patterns	Frequency in ADNI [3] database	Frequency OASIS [4] database
51	{L.mAmyg, L.NAC} ⟶ { #, R.lAmyg}	17.4%	11.1%
52	{L.NAC} ⟶ { #, L.A28/34, L.vmPu}	17.4%	11.1%
53	{L.NAC} ⟶ { #, R.mAmyg ,R.NAC}	17.4%	5.6%
54	{L.vIa, L.mAmyg, L.lAmyg, L.NAC, R.NAC} ⟶ { #, R.A28/34}	17.4%	11.1%
55	{L.vIa, L.mAmyg, L.lAmyg, L.NAC} ⟶ { #, L.rHipp}	17.4%	5.6%
56	{L.TI, L.vIa, L.vId/vIg, L.mAmyg, L.lAmyg} ⟶ { #, L.rHipp}	17.4%	11.1%
57	{L.A38l, L.A35/36r, L.TI, L.vIa, L.vId/vIg, L.mAmyg, R.mAmyg, L.lAmyg, L.NAC} ⟶ { #, L.vmPu}	17.4%	5.6%
58	{L.mAmyg, L.NAC, R.NAC} ⟶ { #, L.vmPu}	16.8%	5.6%
59	{L.lAmyg} ⟶ { #, R.NAC}	16.8%	5.6%
60	{L.NAC} ⟶ { #, L.vId/vIg}	16.8%	16.7%
61	{L.A35/36r, L.TI, L.vIa, L.vId/vIg, L.lAmyg} ⟶ { #, L.rHipp}	16.8%	16.7%
62	{L.A38l, L.TI, L.vIa, L.vId/vIg, L.lAmyg} ⟶ { #, L.rHipp}	16.8%	16.7%
63	{L.lAmyg} ⟶ { #, R.mAmyg}	16.8%	16.7%
64	{L.vIa, L.mAmyg, L.lAmyg, L.NAC, R.NAC} ⟶ { #, L.A28/34}	16.8%	0%
65	{L.vIa, L.mAmyg, R.mAmyg, L.lAmyg, L.NAC, R.NAC} ⟶ { #, R.A28/34}	16.8%	11.1%
66	{L.A38l, L.A35/36r, L.TI, L.vIa, L.vId/vIg, L.mAmyg, R.mAmyg, L.lAmyg, L.rHipp, L.NAC} ⟶ { #, R.A28/34}	16.8%	5.6%
67	{L.lAmyg} ⟶ { #, L.vId/vIg}	16.8%	16.7%
68	{L.lAmyg} ⟶ { #, L.A38l}	16.8%	16.7%
69	{L.mAmyg, L.NAC} ⟶ { #, L.A28/34, L.vmPu}	16.8%	11.1%
70	{L.TI, L.vIa, L.vId/vIg, L.mAmyg, L.lAmyg, L.NAC, R.NAC} ⟶ { #, R.A28/34}	16.8%	11.1%
71	{L.TI, L.vIa, L.mAmyg, L.lAmyg, L.NAC} ⟶ { #, L.rHipp}	16.8%	5.6%
72	{L.A35/36r, L.TI, L.vIa, L.vId/vIg, L.mAmyg, R.mAmyg, L.lAmyg, L.NAC} ⟶ { #, L.A28/34}	16.8%	0%
73	{L.A38l, L.TI, L.vIa, L.vId/vIg, L.mAmyg, R.mAmyg, L.lAmyg, L.NAC} ⟶ { #, L.A28/34}	16.8%	0%
74	{L.vIa, L.lAmyg} ⟶ { #, R.NAC}	16.8%	5.6%
75	{L.NAC} ⟶ { #, L.lAmyg}	16.2%	16.7%
76	{L.NAC} ⟶ { #, L.TI, L.vId/vIg}	16.2%	16.7%
77	{L.NAC} ⟶ { #, L.A28/34, R.A28/34}	16.2%	16.7%
78	{L.vIa, L.mAmyg, L.lAmyg, L.NAC, R.NAC} ⟶ { #, L.vmPu}	16.2%	5.6%
79	{L.vIa, L.lAmyg} ⟶ { #, R.mAmyg}	16.2%	16.7%
80	{L.vIa, L.mAmyg, R.mAmyg, L.lAmyg, L.NAC, R.NAC} ⟶ { #, L.A28/34}	16.2%	0%
81	{L.mAmyg, R.mAmyg, L.NAC, R.NAC} ⟶ { #, L.vmPu}	16.2%	5.6%
82	{L.mAmyg} ⟶ { #, L.TI}	16.7%	13.6%
83	{L.TI, L.vIa, L.vId/vIg, L.mAmyg, R.mAmyg, L.lAmyg, L.NAC, R.NAC} ⟶ { #, R.A28/34}	16.2%	11.1%
84	{L.A38l, L.A35/36r, L.TI, L.vIa, L.vId/vIg, L.mAmyg, L.lAmyg, L.NAC, R.NAC} ⟶ { #, R.A28/34}	16.2%	11.1%
85	{L.A35/36r, L.TI, L.vIa, L.vId/vIg, L.mAmyg, L.lAmyg} ⟶ { #, L.rHipp}	16.2%	11.1%
86	{L.A38l, L.A35/36r, L.TI, L.vIa, L.vId/vIg, L.mAmyg, R.mAmyg, L.lAmyg, L.NAC} ⟶ { #, L.A28/34}	16.2%	0%
87	{L.A38l, L.TI, L.vIa, L.vId/vIg, L.mAmyg, L.lAmyg} ⟶ { #, L.rHipp}	16.2%	11.1%

Symbol # represents the elements in the antecedent.

References

[1] L. Fan, H. Li, J. Zhuo, Y. Zhang, J. Wang, L. Chen, Z. Yang, C. Chu, S. Xie, A. R. Laird, et al., *Cerebral cortex* **2016**, *26*, 8 3508.

[2] B. W. Matthews, *Biochimica et Biophysica Acta (BBA)-Protein Structure* **1975**, *405*, 2 442.

[3] S. G. Mueller, M. W. Weiner, L. J. Thal, R. C. Petersen, C. Jack, W. Jagust, J. Q. Trojanowski, A. W. Toga, L. Beckett, *Neuroimaging Clinics* **2005**, *15*, 4 869.

[4] P. J. LaMontagne, T. L. Benzinger, J. C. Morris, S. Keefe, R. Hornbeck, C. Xiong, E. Grant, J. Hassenstab, K. Moulder, A. G. Vlassenko, et al., *MedRxiv* **2019**.

[5] C. Salvatore, A. Cerasa, P. Battista, M. C. Gilardi, A. Quattrone, I. Castiglioni, *Frontiers in neuroscience* **2015**, *9* 307.

[6] J. Hu, L. Shen, G. Sun, In *Proceedings of the IEEE conference on computer vision and pattern recognition.* **2018** 7132–7141.

[7] C. Lian, M. Liu, J. Zhang, D. Shen, *IEEE transactions on pattern analysis and machine intelligence* **2018**, *42*, 4 880.

[8] R. M. Crum, J. C. Anthony, S. S. Bassett, M. F. Folstein, *Jama* **1993**, *269*, 18 2386.

[9] R. S. Doody, P. Massman, J. K. Dunn, *Archives of neurology* **2001**, *58*, 3 449.

[10] B. Fischl, *Neuroimage* **2012**, *62*, 2 774.

[11] S. Even, *Graph algorithms*, Cambridge University Press, **2011**.

[12] Y. Hou, X. Dan, M. Babbar, Y. Wei, S. G. Hasselbalch, D. L. Croteau, V. A. Bohr, *Nature Reviews Neurology* **2019**, *15*, 10 565.

[13] M. J. Zaki, *Machine learning* **2001**, *42*, 1 31.
